# Methylsteric Effects Enhance Fluorescence in Diphenylfumaronitrile AIEgens

**DOI:** 10.3390/molecules30142898

**Published:** 2025-07-08

**Authors:** Zihao Xu, Wenwen Ma, Yuchen Song, Yu Tian, Fang Hu, Wenbo Wu, Liu Cai

**Affiliations:** 1 Biomaterials Research Center, School of Biomedical Engineering, Southern Medical University, Guangzhou 510515, China; xuzihaogjbh@163.com (Z.X.); 13571826895@163.com (W.M.); songyuchen_99@163.com (Y.S.); 2 Institute of Molecular Aggregation Science, Tianjin University, Tianjin 300072, China; tianjisaima2011@126.com

**Keywords:** aggregation-induced emission, methyl substitution, fluorescence enhancement, fumaronitrile AIEgens, tumor imaging

## Abstract

The development of fluorophores with high-fluorescence quantum yields is highly desirable. To regulate photophysical properties, previous fumaronitrile-core fluorophore designs primarily employed electron-donating structure modifications and π-conjugation extension strategies. Here, we report a novel strategy to enhance the fluorescence performance of fluorophores by introducing methyl groups into fumaronitrile phenyl rings. The introduction of methyl groups reduces the ability to generate reactive oxygen species while enhancing the fluorescence quantum yield. Notably, after encapsulating DSPE-PEG2000 to form nanoparticles, TFN-Me nanoparticles exhibited superior fluorescence performance than previously reported fluorophores and successfully applied in in vivo tumor fluorescence imaging. This study indicates that the methyl introduction strategy holds the potential to become a powerful tool for developing high-brightness fluorophores with fumaronitrile structure.

## 1. Introduction

Fluorescence imaging technology has been widely used for dynamic monitoring and imaging in biological systems [1,2,3,4] owing to its high sensitivity, non-invasiveness, and real-time visualization capabilities [5,6,7]. Nevertheless, conventional organic fluorophores fail to fully meet the demands of modern bioimaging applications. While these probes exhibit excellent emission characteristics in dispersed solutions [8,9], they suffer from aggregation-caused quenching (ACQ) due to non-radiative decay upon aggregation [10], significantly compromising detection performance. On the contrary, fluorophores with aggregation-induced emission (AIE) characteristics demonstrate negligible emissions in the dissolved state; however, they exhibit dramatically enhanced luminescence upon aggregation due to the restriction of intramolecular motion and the inhibition of non-radiative decay [11,12]. The higher the degree of aggregation of AIEgens, the brighter the fluorescence. Based on this characteristic, AIE-based probes have been extensively designed as fluorophores in fluorescence imaging for various applications, such as fluorescence-guided surgery, bacteria detection, in vivo real-time biomarker detection, and so on [13,14,15,16,17,18,19,20].

Most currently available organic fluorophores are limited by low fluorescence quantum yields and short-tissue-penetration wavelengths, which has substantially impeded their imaging performance in vivo [14,21]. When constructing organic long-wavelength emitters, the utilization of doner–acceptor and intramolecular charge transfer are common strategies to achieve absorption and emission red-shift [22,23,24,25,26]. Fumaronitrile (FN) moiety is a classical electron acceptor unit and has been actively incorporated into the structural design of AIEgens for photodynamic therapy (PDT) and fluorescence imaging [27,28,29,30]. Compared to other electron acceptor units, fumaronitrile exhibits proper electron-withdrawing capacity and enhanced ease of modification, making it an ideal candidate for designing high-performance fluorophores.

Fluorescence intensity is a critical determinant of the detection performance of fluorophores. In previous studies, the design of AIEgens based on fumaronitrile moiety has primarily focused on modifications to electron-donating structures, such as the modulation of electronic structure and the extension of π-conjugation [27,31,32,33] by introducing classical electron donors such as tetraphenylethylene (TPE), triphenylamine (TPA), and so on [34,35,36]. Symmetric donor–acceptor–donor (D-A-D)-type structures were constructed to precisely regulate the photophysical properties of AIEgens, including their absorption and fluorescence emission wavelengths, fluorescence intensity, and reactive oxygen species (ROS) generation capability [37,38]. However, this design strategy constrains further molecular optimization, while introducing limitations such as limited fluorescence enhancement, evenly reduced fluorescence in solution or nanoparticles (NPs) [39], and reduced solubility, ultimately impeding broader development and practical applications range.

**Scheme 1:**
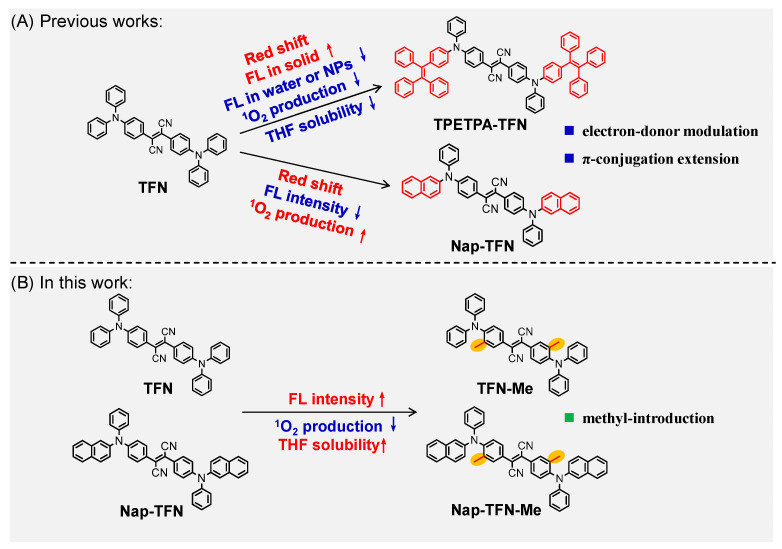
Design strategy of TFN. (**A**) Previous works: electron donor modulation and π-conjugation extension. (**B**) In this work: methyl introduction strategy.

In this regard, we herein report a novel modification strategy for AIEgens based on the fumaronitrile scaffold. Through targeted methyl substitution on the fumaronitrile phenyl ring, we successfully synthesized two new D-A-D structure-based AIEgens, TFN-Me and Nap-TFN-Me (Scheme 1). Remarkably, these methylated probes demonstrate significantly enhanced fluorescence intensity compared to their non-methylated counterparts, but reduced singlet oxygen (^1^O_2_) generation capability. The AIEgens were then encapsulated into NPs using DSPE-PEG2000 (Shanghai Yuanye Bio-Technology Co., Ltd., Shanghai, China), followed by in vitro evaluation of their physicochemical properties, including singlet oxygen generation efficiency, cell cytotoxicity, and fluorescence intensity. Furthermore, the fluorescence imaging performance of NPs in vivo was also evaluated in 4T1 tumor-bearing mice.

## 2. Results and Discussion

### 2.1. Synthesis and Characterization of Fluorophores

**Figure 1:**
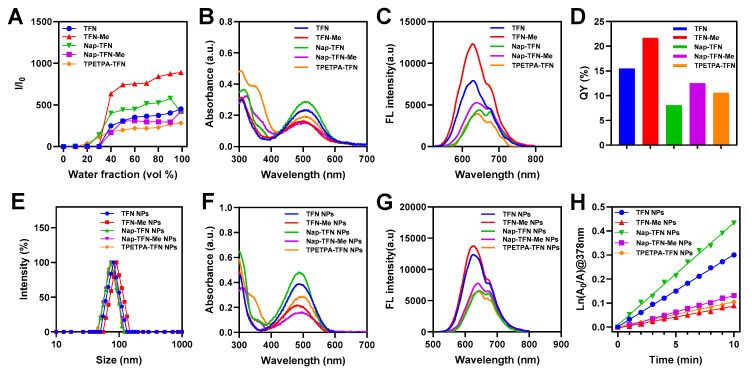
(**A**) The plot of relative maximum emission intensities of TFN, TFN-Me, Nap-TFN, Nap-TFN-Me and TPETPA-TFN (10 μM) in different DMSO/water mixtures. Absorption (**B**) and fluorescence (**C**) spectra of TFN, TFN-Me, Nap-TFN, Nap-TFN-Me, and TPETPA-TFN (10 μM) in the DMSO/water (1/99, *v*/*v*) solution. (**D**) Fluorescence quantum yield of TFN, TFN-Me, Nap-TFN, Nap-TFN-Me, and TPETPA-TFN. Hydrodynamic size distribution (**E**), absorption (**F**) and fluorescence (**G**) spectra of TFN NPs, TFN-Me NPs, Nap-TFN NPs, Nap-TFN-Me NPs, and TPETPA-TFN NPs in water solution. (**H**) The degradation rates of ABDA by TFN NPs, TFN-Me NPs, Nap-TFN NPs, Nap-TFN-Me NPs, and TPETPA-TFN NPs under white light irradiation (50 mW/cm^2^), where A_0_ and A are the absorbance of ABDA at 378 nm at 0 min and 0–10 min, respectively.

The non-radiative decay caused by small energy gaps is an important reason to hinder the high-fluorescence quantum yield [40]. According to the previously reported literature, the introduction of large-volume units within a confined space will induce significant repulsive interactions, which effectively suppresses non-radiative transition and restricts the rotation of dihedral angles in the excited state, thereby enhancing fluorescence performance [15]. Methyl is considered as a bulky steric hindrance unit. Based on this molecular design strategy, TFN, TFN-Me, Nap-TFN, Nap-TFN-Me and TPETPA-TFN were synthesized using diphenylamine derivative and we synthesized 2,3-bis(4-bromophenyl)-2-butenedinitrile derivative via a Buchwald–Hartwig cross-coupling reaction. The synthetic routes of the fluorophores above are shown in Scheme S1. The chemical structures were confirmed by ^1^H NMR, ^13^C NMR, and mass spectrum analysis (Figures S1–S12). These probes are composed of an AIE electron-donating core and a fumaronitrile electron-accepting scaffold. They comprise a D-A-D structure, which endows them with red emissions. The optical properties of fluorophores are shown in Figure 1. The AIE properties of TFN, TFN-Me, Nap-TFN, Nap-TFN-Me, and TPETPA-TFN were evaluated in different DMSO/water solutions (Figure S13). As shown in Figure 1A and Figure S14, all five fluorophores exhibited negligible luminescence in DMSO solutions; however, their fluorescence intensity progressively increased when the water fraction exceeded 40% and continued to rise with further aqueous content, exhibiting distinct AIE characteristics. Notably, when the water fraction increased from 90% to 99%, Nap-TFN exhibited a marked decrease in fluorescence intensity, attributable to a characteristic twisted intramolecular charge transfer effect.

The maximum absorption wavelengths were determined to be 505 nm for TFN, 497 nm for TFN-Me, 510 nm for Nap-TFN, 500 nm for Nap-TFN-Me and 508 nm for TPETPA-TFN in water (Figure 1B), and the absorption spectra of them in DMSO were shown in Figure S15. The maximum fluorescence emission wavelengths in water are 629 nm for TFN, 626 nm for TFN-Me, 646 nm for Nap-TFN, 639 nm for Nap-TFN-Me, and 642 nm for TPETPA-TFN, respectively (Figure 1C). By replacing phenyl with naphthyl and tetraphenylethenyl, the maximum absorption and fluorescence emission peaks of Nap-TFN and TPETPA-TFN both show red shift compared with TFN. The introduction of methyl into 2,3-bis(4-bromophenyl)-2-butenedinitrile derivative structure can twist the D-A-D structure and may led to the blue-shifted absorption and fluorescence emission peaks. Fortunately, the blue-shifts are slight according to the above measurement. Especially, TFN-Me has higher fluorescence intensity than TFN in water, likewise, the fluorescence intensity of Nap-TFN-Me is higher than Nap-TFN. TFN-Me exhibited 1.56-fold higher fluorescence intensity than TFN, while Nap-TFN-Me showed a 1.2-fold enhancement compared to Nap-TFN. These indicate that the introduction of methyl is a strategy for these fluorophores to enhance fluorescence efficiency. By using 4-(dicyanomethylene)-6-[4-(dimethylamino)styryl]-2-methyl-4H-pyran (DCM) as a reference, the fluorescence quantum yield of TFN, TFN-Me, Nap-TFN, Nap-TFN-Me, and TPETPA-TFN aggregates in water were measured and calculated to be 15.5%, 21.7%, 8.1%, 12.5% and 10.6% (Figure 1D, Figures S16 and S17), respectively. TFN-Me exhibited a 1.4-fold higher fluorescence quantum yield than TFN, while Nap-TFN-Me showed a 1.54-fold increase relative to Nap-TFN. TPETPA-TFN, a previously reported AIE fluorophore, demonstrates excellent fluorescence quantum yield and has been successfully implemented in multiple applications, including solar thin polymer films, in vivo bioimaging, and organic light-emitting diodes (OLEDs) [39,41,42,43]. However, the fluorescence performance of TPETPA-TFN aggregate in water or NPs is not as good. These results also confirm that methyl introduction strategy is beneficial to enhance the fluorescence efficiency of fluorophores in aggregate or NPs form, due to the repulsive interactions resulting from the methyl units, non-radiative transition was suppressed effectively.

### 2.2. Preparation and Characterization of NPs

To increase water dispersibility, simulate, and evaluate the intracellular performance [44], we utilized DSPE-PEG2000 as the carrier to form NPs by a nanoprecipitation method with the particle size of ≈80 nm (Figure 1E and Figure S18). Suitable nanostructures and sizes, especially 50–200 nm [45], are profit for the progressive accumulation of NPs in tumor regions over time through the enhanced permeability and retention (EPR) effect, resulting in passive targeting [46]. All five NPs exhibited comparable negative zeta potentials in water solution (Figure S19). The NPs had even sizes and exhibited excellent structure stability in water during 7 days’ storage (Figures S20 and S21), ensuring reliable performance for both follow-up in vitro and in vivo applications. The maximum absorption wavelengths were determined to be 488 nm for TFN NPs, 482 nm for TFN-Me NPs, 490 nm for Nap-TFN NPs, 486 nm for Nap-TFN-Me NPs, and 497 nm for TPETPA-TFN NPs (Figure 1F), and the maximum fluorescence emission peaks of them are 630 nm, 625 nm, 643 nm, 640 nm, and 642 nm, respectively (Figure 1G). The fluorophore-loaded nanoparticles exhibited a ≈ 15 nm blue shift in absorption wavelength compared to the free fluorophore in water. Notably, TFN-Me NPs exhibited reduced absorbance value relative to TFN NPs, with Nap-TFN-Me showing a similar decreasing trend versus Nap-TFN NPs. The methyl introduction strategy for fluorescence enhancement remains effective in nanoparticle systems. As shown in Figure 1G, TFN-Me NPs exhibited 1.12-fold enhanced fluorescence intensity compared to TFN NPs, while Nap-TFN-Me NPs demonstrated a 1.19-fold increase relative to Nap-TFN NPs. The fluorescence quantum yields of TFN NPs, TFN-Me NPs, Nap-TFN NPs, Nap-TFN-Me NPs, and TPETPA-TFN NPs were measured and calculated to be 14.5%, 29.1%, 8.3%, 22%, and 10.3%, respectively. The optical properties of NPs are like those of aggregates in water.

### 2.3. Photophysical Performance of NPs In Vitro

Then we evaluated the singlet oxygen (^1^O_2_) generation performance of these five NPs. 9,10-anthracenediyl-bis(methylene)dimalonic acid (ABDA) is a commonly used singlet oxygen detection probe [47]. When ABDA reacts with singlet oxygen, the anthracene ring structure will react into an internal peroxide, and the amount of generated internal peroxide is proportional to the amount of singlet oxygen reacting with ABDA. The generated internal peroxide will cause a decrease in the absorbance of ABDA at 378 nm. As shown in Figure 1H and Figure S22, the rank of singlet oxygen generation capacity is Nap-TFN NPs > TFN NPs > Nap-TFN-Me NPs > TPETPA-TFN NPs > TFN-Me NPs. Using ABDA degradation kinetics as the quantitative metric, the singlet oxygen generation capacity of TFN-Me NPs decreased to 33.8% of TFN NPs, while Nap-TFN-Me NPs showed 35% relative to Nap-TFN NPs, demonstrate that the introduction of methyl substantially damages singlet oxygen production capacity, with an observed reduction of 65–66.2% relative to non-methylated analogs based on the slope in Figure 1H. Contrary to the result of fluorescence intensity, the stronger the fluorescence intensity is, the less singlet oxygen is generated. The above results suggest that methyl introduction strategy severely reduces the ability to generate singlet oxygen, which is beneficial for imaging with lower photo-toxicity.

**Figure 2:**
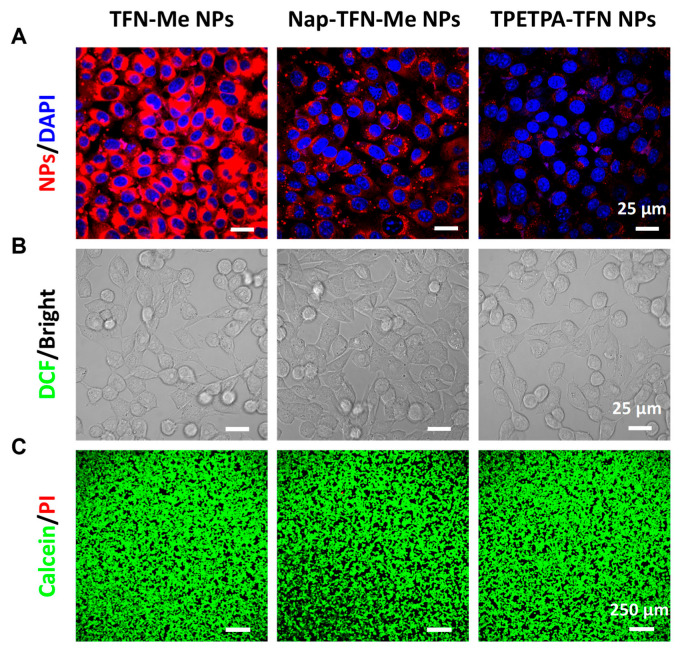
(**A**) CLSM merge images of endocytosis of 4T1 cells after co-incubation of TFN-Me NPs, Nap-TFN-Me NPs, and TPETPA-TFN NPs (50 μg/mL) for 8 h. Scale bars = 25 µm. (**B**) 4T1 cells were incubated with TFN-Me NPs, Nap-TFN-Me NPs and TPETPA-TFN NPs (50 μg/mL) for 8 h, and the ROS generation observed by CLSM. Scale bars = 25 µm. (**C**) 4T1 cells were incubated with TFN-Me NPs, Nap-TFN-Me NPs and TPETPA-TFN NPs (50 μg/mL) for 8 h, and the live and dead cells were observed by CLSM. Scale bars = 250 µm.

TPETPA-TFN NPs were mostly used in cell imaging [31,39]. To evaluate the fluorescence performance of methyl-based fluorophores NPs in cells, the intracellular distribution and fluorescence characteristics of TFN-Me NPs, Nap-TFN-Me NPs were observed by confocal laser scanning microscopy (CLSM), to compare with TPETPA-TFN NPs. As shown in Figure 2A, in NP-treated 4T1 cells, the red fluorescence intensity in the cytoplasm gradually increased with prolonged incubation time, reaching its peak at 8 h. After 8 h of co-incubation, both TFN-Me NPs and Nap-TFN-Me NPs exhibited significantly stronger intracellular fluorescence intensity compared to TPETPA-TFN NPs. The fluorescence intensity is TFN-Me NPs > Nap-TFN-Me NPs > TPETPA-TFN NPs. These were consistent with the results in the extracellular environment. These results indicated that TFN-Me possessed superior fluorescence performance and could be used as a fluorescent probe for in vitro bioimaging.

The intracellular ROS generation levels induced by NPs were evaluated using 2′,7′-dichlorodihydrofluorescein diacetate (DCFH-DA) as a fluorescent probe. DCFH-DA is intrinsically non-fluorescent, upon cellular uptake, its acetate groups are hydrolyzed by intracellular esterases to yield DCFH, which is subsequently oxidized by ROS into green-fluorescent DCF. The resulting green fluorescence intensity directly correlates with intracellular ROS levels. As shown in Figure 2B, after 8 h of co-incubation with 4T1 cells, no obvious fluorescence signal was observed in all three NPs groups in dark control conditions. The negligible levels of ROS generation demonstrated that the NPs exhibited minimal dark toxicity and excellent biosafety profiles, which make them highly suitable for in vivo biological applications.

To further evaluate the cytotoxic efficacy of NPs against 4T1 cells, a live/dead assay was performed by using Calcein-AM and propidium iodide (PI). Calcein-AM freely permeates intact plasma membranes of viable cells, where intracellular esterases hydrolyze them into Calcein, which exhibits intensely green fluorescence under 490 nm laser excitation; the fluorescence intensity is directly proportional to cellular metabolic activity. Conversely, when membrane integrity is compromised, propidium iodide (PI) enters cells and intercalates with nucleic acids, emitting red fluorescence upon 535 nm laser excitation [48]. As shown in Figure 2C, following 8 h of co-incubation with NPs, all three NPs groups exhibited only green fluorescence signals, with almost no appreciable red fluorescence observed, indicating that most cells remained viable after being treated with NPs, confirming their negligible dark toxicity toward 4T1 cells.

**Figure 3:**
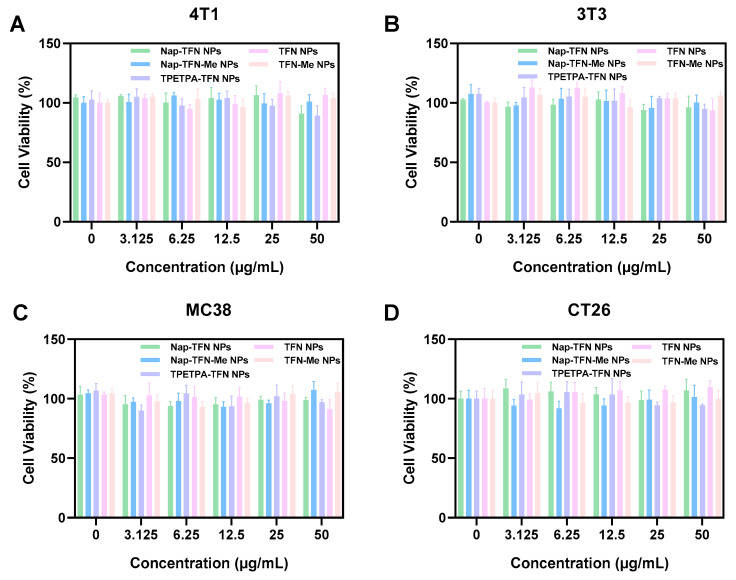
Cell viabilities of different concentrations of TFN NPs, TFN-Me NPs, Nap-TFN NPs, Nap-TFN-Me NPs, and TPETPA-TFN NPs after 24 h of co-incubation with 4T1 cells (**A**), 3T3 cells (**B**), MC38 cells (**C**), and CT26 cells (**D**) in darkness. Error bars: mean ± SD (n = 5).

Afterward, to further evaluate the potential dark toxicity of NPs towards cells, cell viability was quantified using the MTT colorimetric assay, which is based on the reduction in yellow 3-(4,5-dimethylthiazol-2-yl)-2,5-diphenyltetrazolium bromide (MTT) to purple formazan crystals by mitochondrial succinate dehydrogenase in metabolically active cells. 4T1 cells, MC38 cells, and CT26 cells are all murine-derived cancer cells, while 3T3 cells are normal murine cells. As shown in Figure 3, no significant difference in cell viabilities were observed across all four types of cells at the 0–50 μg/mL concentration range, even when incubated with NPs at a high concentration of 50 μg/mL under dark conditions. All four types of cells maintained high viability rates (>90%), demonstrating the low toxicity and favorable biocompatibility of the NPs towards both cancer cells and normal cells. In addition, the phototoxicity of NPs towards 4T1 cells was also evaluated via MTT assay. As depicted in Figure S23, upon 100 mW/cm^2^ white light irradiation, TFN NPs, TFN-Me NPs, Nap-TFN-M,e and TPETPA-TFN NPs exhibited limited phototoxicity towards 4T1 cells, less than 15% cell death even at 50 μg/mL because of the low ^1^O_2_ production capacities, whereas Nap-TFN NPs demonstrated enhanced phototoxicity. Moreover, the 4T1 cell images observed by microscope revealed relatively intact cell structures and sufficient cell densities, further confirming the minimal phototoxicities (Figure S24). These results match well with the solution level measurements.

Above all, the combined results from ROS assay, live/dead staining, and MTT analyses consistently demonstrate that TFN NPs, TFN-Me NPs, Nap-TFN NPs, Nap-TFN-Me NPs, and TPETPA-TFN NPs both exhibit low cytotoxicity under dark condition and low cell-killing capacity upon irradiation. Hence, these NPs can be used as excellent fluorophores with low side effects.

### 2.4. Fluorescence Imaging of Tumor Cells In Vivo

**Figure 4:**
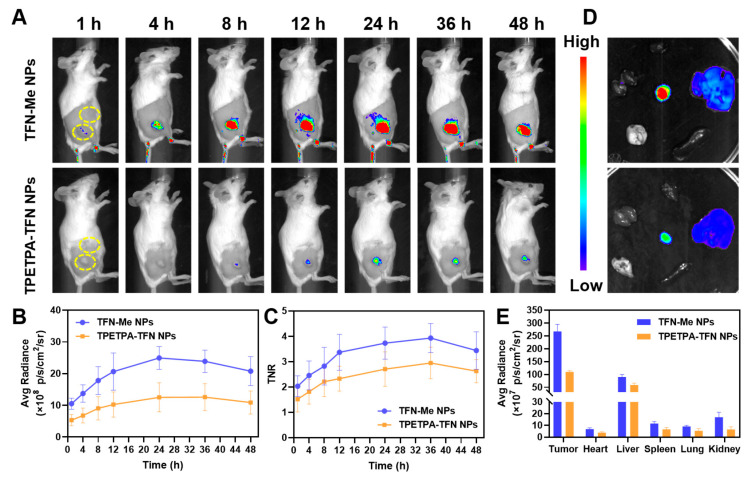
(**A**) Fluorescence images of intravenous injection of TFN-Me NPs and TPETPA-TFN NPs (5 mg/kg) into 4T1 subcutaneous tumor-bearing mice for 1, 4, 8, 12, 24, 36, and 48 h. Change in fluorescence intensity (**B**) and tumor normal ratio (TNR) (**C**) of tumor regions at different times after injection of TFN-Me NPs and TPETPA-TFN NPs. Fluorescence images of tumor and major organs excised from mice treated with TFN-Me NPs and TPETPA-TFN NPs (**D**) and quantitative analysis of fluorescence intensity (**E**) after intravenous injection at 48 h. Error bars: mean ± SD (n = 3). The TNR analysis regions were demarcated by the yellow dashed ellipse in (**A**).

Encouraged by the good performance of TFN-Me in vitro, we further verified the fluorescence imaging performance of TFN-Me NPs in vivo. Before conducting in vivo experiments, the hemocompatibility of NPs was systematically evaluated through hemolysis assays, demonstrating excellent blood compatibility with <5% hemolysis rate at clinically relevant concentrations (Figure S25). TFN-Me NPs and TPETPA-TFN NPs were intravenously injected into the 4T1 tumor-bearing mice. After the injection of NPs, the fluorescence imaging was obtained at different times. It is obvious that two NPs gradually accumulated at the tumor regions, demonstrating that the nanoparticles effectively accumulate in tumor regions and achieve passive retention through the enhanced permeability and retention effect. As shown in Figure 4A, the fluorescence intensities of TFN-Me NPs and TPETPA-TFN NPs in tumor regions exhibited a time-dependent increase, reaching a peak at 24 h (Figure 4B), and then declined gradually. Moreover, the fluorescence intensity of TFN-Me NPs group at tumor regions was significantly stronger than TPETPA-TFN NPs group, indicating that TFN-Me NPs have exceptional in vivo tumor imaging capabilities. The peak tumor normal ratio (TNR) was achieved at 36 h post-injection (Figure 4C). At 48 h of post-administration, the mice were sacrificed, and tumors along with major organs were harvested for ex vivo imaging and analysis. As depicted in Figure 4D,E, the fluorescence signals revealed that the NPs administered via intravenous injection exhibited predominant accumulation in tumor regions with secondary hepatic distribution. Afterward, cardiac, hepatic, and renal functions were comprehensively evaluated using six serum biomarkers, biochemical analysis confirmed that both TFN-Me NPs and TPETPA-TFN NPs groups maintained all serum parameters within the normal range (Figure S26). Additionally, the histological assessment by hematoxylin and eosin (H&E) staining sections of major normal organs including the heart, liver, spleen, lung, and kidney revealed intact tissue architecture with no signs of damage (Figure S27), further demonstrating the high biocompatibility of NPs.

## 3. Materials and Methods

The synthesis of TFN-Me is as follows. In a 300 mL round-bottom flask, 2,3-bis(4-bromo-3-methylphenyl)fumaronitrile (200 mg, 0.48 mmol), diphenylamine (244 mg, 1.44 mmol), Cs_2_CO_3_ (400 mg, 1.2 mmol), Pd_2_(dba)_3_ (22 mg, 0.02 mmol), and Ruphos (24 mg, 0.04 mmol) were dissolved in 15 mL toluene under N_2_ protection; then, the mixture was heated to 110 °C and stirred for 24 h. Upon completion, toluene was removed by rotary evaporation (Shanghai Ailang Instrument Co., Ltd., Shanghai, China) under reduced pressure. The residue was extracted with dichloromethane and brine; the organic layer was collected, dried over anhydrous sodium sulfate and concentrated by evaporation. The crude product was purified by column chromatography on silica gel with petroleum ether/dichloromethane (5:1, *v*/*v*) to obtain TFN-Me as a red solid, 66% yield: ^1^H NMR (400 MHz, CDCl_3_) δ 7.61 (dt, *J* = 8.4, 7.3 Hz, 4H), 7.18 (t, *J* = 7.9 Hz, 8H), 7.12 (d, *J* = 8.3 Hz, 2H), 6.93 (dd, *J* = 13.3, 7.5 Hz, 12H), 1.97 (s, 6H). ^13^C NMR (101 MHz, CDCl_3_) δ 149.28 (s), 147.35 (s), 136.27 (s), 132.51 (s), 129.48 (s), 129.02 (s), 128.77 (s), 127.81 (s), 123.21 (s), 122.94 (d, *J* = 12.4 Hz), 117.35 (s), 19.36 (s). ESI-MS, *m*/*z*: [M + H^+^] calcd for C_42_H_33_N_4_ 593.26, found 563.23.

The synthesis of Nap-TFN-Me is as follows. In a 300 mL round-bottom flask, 2,3-bis(4-bromo-3-methylphenyl)fumaronitrile (100 mg, 0.24 mmol), *N*-phenyl-2-naphthylamine (132 mg, 0.6 mmol), Cs_2_CO_3_ (200 mg, 0.6 mmol), Pd_2_(dba)_3_ (11 mg, 0.01 mmol), and Ruphos (12 mg, 0.02 mmol) were dissolved in 15 mL toluene under N_2_ protection; then, the mixture was heated to 110 °C and stirred for 24 h. Upon completion, toluene was removed by rotary evaporation under reduced pressure. The residue was extracted with dichloromethane and brine; the organic layer was collected, dried over anhydrous sodium sulfate, and concentrated by evaporation. The crude product was purified by column chromatography on silica gel with petroleum ether/dichloromethane (5:1, *v*/*v*) to obtain Nap-TFN-Me as a red solid, with a 71% yield: ^1^H NMR (400 MHz, CDCl_3_) δ 7.80–7.67 (m, 8H), 7.61 (d, *J* = 7.9 Hz, 2H), 7.46–7.28 (m, 8H), 7.23 (s, 4H), 7.06 (t, *J* = 8.3 Hz, 6H), 2.06 (s, 6H). ^13^C NMR (101 MHz, CDCl_3_) δ 149.24 (s), 147.31 (s), 144.88 (s), 136.25 (s), 134.49 (s), 132.59 (s), 130.14 (s), 129.57 (s), 129.33–128.72 (m), 127.82 (d, *J* = 14.5 Hz), 127.12 (s), 126.64 (s), 124.78 (s), 123.30 (t, *J* = 9.4 Hz), 119.04 (s), 117.37 (s), 19.42 (s). ESI-MS, *m*/*z*: [M + H^+^] calcd for C_50_H_37_N_4_ 693.29, found 693.27.

The preparation of nanoparticles is as follows. TFN, TFN-Me, Nap-TFN, Nap-TFN-Me, and TPETPA-TFN (1 mg) were dissolved in THF (1 mL) and then mixed separately with DSPE-PEG2000 (5 mg); after that, the mixture solution was injected into water (10 mL) under ultrasonic (Ningbo Scientz Biotechnology Co., Ltd., Ningbo, China) condition for 3 min. Upon completion, THF was volatilized by stirring at room temperature for 16 h. The obtained nanoparticles were filtered through 0.22 μm PES syringe filter and stored in refrigerator at 4 °C for later use.

Singlet oxygen generation measurement is as follows. The singlet oxygen (^1^O_2_) generation ability of nanoparticles was measured by using ABDA through UV-vis absorption spectrum. The nanoparticles (1 mM, 10 μL) and ABDA (10 mM, 10 μL) were mixed and dissolved in water (980 μL), and then irradiated with white light (50 mW/cm^2^), the absorbance value at the wavelength of 378 nm of ABDA was measured every 1 min.

Fluorescence quantum yield measurement is as follows. The fluorescence quantum yield (*Φ*_f_) of TFN, TFN-Me, Nap-TFN, Nap-TFN-Me, and TPETPA-TFN were determined by reference method. DCM was used as reference, which has known a fluorescence quantum yield (*Φ*_DCM_ = 43.5% in methanol). The UV-vis absorption spectra of TFN, TFN-Me, Nap-TFN, Nap-TFN-Me, TPETPA-TFN, and DCM were measured. The absorbance values at the maximum absorption peak should be maintained below 0.1 to prevent fluorescence signal inaccuracy caused by self-absorption and inner filter effects at high concentrations. Then we measured the fluorescence spectrum excited by maximum absorption wavelength. The fluorescence quantum yields (*Φ*_f_) of TFN, TFN-Me, Nap-TFN, Nap-TFN-Me, and TPETPA-TFN were calculated by the following formula:

$$\Phi_{f}=\Phi_{DCM}(\frac{k_{f}}{k_{DCM}})(\frac{\eta_{f}^{2}}{\eta_{DCM}^{2}})$$

where *Φ*_DCM_ is the standard fluorescence quantum yield (43.5%) of DCM in methanol. *k*_f_ and *k*_DCM_ are the slope of the linear regression fitting curve between fluorescence intensity integral and absorbance value, respectively. *η*_f_ and *η*_DCM_ are refractive index of water and methanol, respectively.

## 4. Conclusions

In summary, we proposed a novel methyl introduction strategy to enhance the fluorescence performance of AIEgens. After the introduction of the methyl into phenyl, the absorption peaks of generated TFN-Me and Nap-TFN-Me were only slightly blue-shifted, by 8 nm and 10 nm, respectively. Surprisingly, both TFN-Me and Nap-TFN-Me exhibited fluorescence enhancement compared to their non-methylated counterparts, and the quantum yields of the AIEgens were significantly improved, with fluorescence quantum yields of 21.7% and 12.5%, respectively. Furthermore, the methylated AIEgens exhibited dramatically reduced singlet oxygen generation capacity, consequently demonstrating minimal photo/dark toxicity, which is beneficial for in vivo fluorescence imaging applications. Finally, following the performance of the AIEgens, we successfully achieved in vivo tumor fluorescence imaging in mice, demonstrating a superior imaging performance than previously reported AIEgens. We believe that this methyl introduction strategy provides a reliable approach for the development of new high-brightness fluorophores.

## Data Availability

The original contributions presented in this study are included in the article/Supplementary Material. Further inquiries can be directed to the corresponding author(s).

## Supplementary Materials

The following supporting information can be downloaded at: https://www.mdpi.com/article/10.3390/molecules30142898/s1, Scheme S1. The synthetic routes to TFN, TFN-Me, Nap-TFN, Nap-TFN-Me and TPETPA-TFN. Figure S1. 1H NMR spectrum of TFN in CDCl3. Figure S2. 1H NMR spectrum of TFN-Me in CDCl3. Figure S3. 13C NMR spectrum of TFN-Me in CDCl3. Figure S4. 1H NMR spectrum of Nap-TFN in CDCl3. Figure S5. 1H NMR spectrum of Nap-TFN-Me in CDCl3. Figure S6. 13C NMR spectrum of Nap-TFN-Me in CDCl3. Figure S7. 1H NMR spectrum of TPETPA-TFN in CDCl3. Figure S8. Mass spectrum of TFN. Figure S9. Mass spectrum of TFN-Me. Figure S10. Mass spectrum of Nap-TFN. Figure S11. Mass spectrum of Nap-TFN-Me. Figure S12. Mass spectrum of TPETPA-TFN. Figure S13. Fluorescence spectra of TFN (A), TFN-Me (B), Nap-TFN (C), Nap-TFN-Me (D) and TPETPA-TFN (E) (10 μM) in different DMSO/water mixtures. Figure S14. The fluorescence photographs of TFN (A), TFN-Me (B), Nap-TFN (C), Nap-TFN-Me (D) and TPETPA-TFN (E) in DMSO/water mixtures with different water contents under UV light irradiation. λex = 365 nm. Figure S15. Absorption spectra of TFN, TFN-Me, Nap-TFN, Nap-TFN-Me and TPETPA-TFN (10 μM) in DMSO solution. Figure S16. Fluorescence spectra of DCM (A) in methanol, TFN (B), TFN-Me (C), Nap-TFN (D), Nap-TFN-Me (E) and TPETPA-TFN (F) in the DMSO/water (1/99, v/v) solution with different absorbance values. Figure S17. The relationship between the absorbance value and the peak area of fluorescence spectrum measured by DCM (A), TFN (B), TFN-Me (C), Nap-TFN (D), Nap-TFN-Me (E) and TPETPA-TFN (F), and their linear regression fitting curve, respectively. Figure S18. Individual hydrodynamic size distribution and the TEM images (inset photos) of TFN NPs (A), TFN-Me NPs (B), Nap-TFN NPs (C), Nap-TFN-Me NPs (D) and TPETPA-TFN NPs (E). Figure S19. The zeta potential of TFN NPs, TFN-Me NPs, Nap-TFN NPs, Nap-TFN-Me NPs and TPETPA-TFN NPs in water. Error bars: mean ± SD (n = 3). Figure S20. The size changes of TFN NPs (A), TFN-Me NPs (B), Nap-TFN NPs (C), Nap-TFN-Me NPs (D) and TPETPA-TFN NPs (E) within seven storage days, measured by DLS. Error bars: mean ± SD (n = 3). Figure S21. The zeta potential changes of TFN NPs (A), TFN-Me NPs (B), Nap-TFN NPs (C), Nap-TFN-Me NPs (D) and TPETPA-TFN NPs (E) within seven storage days. Error bars: mean ± SD (n = 3). Figure S22. Absorption spectra of mixed solutions of ABDA (100 μM) with TFN NPs (A), TFN-Me NPs (B), Nap-TFN NPs (C), Nap-TFN-Me NPs (D) and TPETPA-TFN NPs (E) in water under 50mW/cm2 white light irradiation at different times. Figure S23. 4T1 cell viabilities after treating with different concentrations of TFN NPs, TFN-Me NPs, Nap-TFN NPs, Nap-TFN-Me NPs and TPETPA-TFN NPs upon white light irradiation (100 mW/cm2) for 5 min. Error bars: mean ± SD (n = 5). Figure S24. The 4T1 cell images observed by microscope upon treatment with TFN NPs, TFN-Me NPs, Nap-TFN NPs, Nap-TFN-Me NPs and TPETPA-TFN NPs (100 μg/mL). Figure S25. (A) Hemolysis rate of red blood cells after being treated with TFN-Me NPs and TPETPA-TFN NPs at a different concentration from 0 to 50 µg/mL for 3 h at 37 °C, using Triton X-100 as a positive control and nanoparticles in PBS without RBCs as a negative control. Error bars: mean ± SD (n = 4). (B) The quantified analysis. Figure S26. Blood biochemistry of mice on ten days post-injection of PBS, TFN-Me NPs and TPETPA-TFN NPs: alanine aminotransferase (ALT) (A), aspartate aminotransferase (AST) (B), blood urea nitrogen (BUN) (C), uric acid (UA) (D), creatine kinase (CK) (E), lactate dehydrogenase (LDH) (F). Error bars: mean ± SD (n = 3). Figure S27. H&E staining of normal organ tissue sections of different groups of mice after treatment.
