# Supplementary material for: Methylsteric Effects Enhance Fluorescence in Diphenylfumaronitrile AIEgens
Source: Molecules. 2025 Jul 8;30(14):2898. doi: 10.3390/molecules30142898 (PMC12300520; doi:10.3390/molecules30142898)
Supplement: Supplementary file 1 [file molecules-30-02898-s001.zip › molecules-3707582-supplementary.pdf]

Methylsteric Effects Enhance Fluorescence in Diphenylfumaronitrile AIEgens

Zihao Xu [1,†], Wenwen Ma [1,†], Yuchen Song [1], Yu Tian [2], Fang Hu [1,*], Wenbo Wu [2,*] and Liu Cai [1,*]

[1] Biomaterials Research Center, School of Biomedical Engineering, Southern Medical University, Guangzhou 510515, China; xuzihaogjbh@163.com (Z.X.); 13571826895@163.com (W.M.); songyuchen_99@163.com (Y.S.)

[2] Institute of Molecular Aggregation Science, Tianjin University, Tianjin 300072, China; tianjisaima2011@126.com

* Correspondence: hufang19@smu.edu.cn (F.H.); wuwb@tju.edu.cn (W.W.); caiiu666666@smu.edu.cn (L.C.)

† These authors contributed equally to this work.

Materials and instruments

Materials. 1-(4-Bromophenyl)-1,2,2-triphenylethene, Xphos, 4-bromo-3-methylbenzeneacetonitrile, $Pd_2(dba)_3$, Cs_2CO_3 and Ruphos were purchased from Bidepharm; aniline, $Pd(OAc)_2$, t-BuONa, sodium methoxide, iodine, diphenylamine, N-phenyl-2-naphthylamine and 2',7'-dichlorodihydrofluorescein diacetate (DCFH-DA) were purchased from Macklin; 4-(dicyanomethylene)-6-[4-(dimethylamino)styryl]-2-methyl-4H-pyran was purchased from Meryer; Chloroform-*d* was pruchased from J&K Scientific; 9,10-anthracenediyl-bis(methylene)dimalonic acid (ABDA) was purchased from Sigma; DSPE-PEG2000 was purchased from Yuanye biotechnology; 3-(4,5-dimethylthiazol-2-yl)-2,5-diphenyltetrazolium bromide (MTT) and Calcein-AM/PI cell double staining kit were purchased from Solarbio; Phosphate-buffered saline (PBS), Dulbecco's modified Eagle medium (DMEM), Roswell Park memorial institute (RPMI−1640) medium, fetal bovine serum origin (FBS), and Penicillin−Streptomycin liquid were purchased from Gibco. All other chemicals were obtained from commercial sources and used as received without further purification.

Instruments: The extent of reaction was monitored by thin-layer chromatography (TLC) using silica gel plates with fluorescent indicators UV254 and UV365 after the plates were subjected to elution in the TLC chamber. Flash column chromatography was carried out using Rhawn silica gel (200−300 mesh). ^{1}H NMR and ^{13}C NMR spectra were measured by the Bruker AVANCE IIITM HD 400MHz nuclear magnetic resonance spectrometer. The mass spectra were measured by Thermo Fisher TSQ Quantiva TM mass spectrometer. The UV-vis absorption spectra were measured on Thermo Fisher Evolution 300 spectrophotometer. Fluorescence spectra were measured by Thermo Fisher Lumina spectrophotometer. The size (diameter, nm) of NPs were measured by the dynamic light scattering (DLS, Brookhaven Nanobrook 90Plus Zeta, USA). The serum biochemical index was detected by Chemray 240 automatic biochemical analyzer. The results of MTT assays were measured by BioTek H1M multi-functional microplate reader to quantify the absorbance of formazan in DMSO solution. Confocal Laser Scanning microscopy images were recorded on a confocal

laser scanning microscope (CLSM, Nikon−A1 HD25). In vivo fluorescence imaging of mice were acquired by PerkinElmer IVIS Lumina III imaging system.

Hydrated particle size of nanoparticles measurement: The hydrated particle size and polydispersity index (PDI) of nanoparticles were measured by dynamic light scattering (DLS).

The stability of nanoparticles: The nanoparticles in pure water were in dark at 4°C, and the average diameter, PDI of which were monitored in seven days.

Cell cultures: The murine breast cancer cells (4T1) were cultured in RPMI-1640 medium supplemented with 10% fetal bovine serum (FBS) and 1% penicillin-streptomycin, under standard incubator conditions (37°C, 5% CO_2). Prior to experiments, the cells were passaged and refreshed with medium until reaching the desired density.

Cell endocytosis: The endocytosis of TFN-Me NPs, Nap-TFN-Me NPs and TPETPA-TFN NPs by 4T1 cells was investigated using a Confocal Laser Scanning Microscope (CLSM). First, 4T1 cells in the logarithmic growth phase were digested. Then, the cells were seeded into confocal dishes at a density of 1×10^5 cells/well and cultured overnight in an incubator at 37°C with a 5% CO_2 concentration to allow them to adhere completely. Next, the 4T1 cells were co - incubated with a culture medium solution containing TFN-Me NPs, Nap-TFN-Me NPs or TPETPA-TFN NPs for 4 hours. After that, the culture medium was aspirated, and the cells were rinsed three times with PBS. Subsequently, 1 mL of 4% paraformaldehyde was added to fix the cells at room temperature for 15 min, followed by three rinses with PBS. Finally, 0.5 mL of 4,6-diamidino-2-phenylindole dihydrochloride (DAPI, 10 μg/mL) was added to stain the cell nuclei for 10 minutes. After three more rinses with PBS, the fluorescence inside the cells was observed using CLSM. The fluorescence of DAPI was excited using a 405 nm channel and displayed in blue, while NPs were excited using a 488 nm channel and displayed in red. This was done to detect the endocytosis of the three fluorescent probes, TFN-Me NPs, Nap-TFN-Me NPs and TPETPA-TFN NPs in 4T1 cells.

Intracellular reactive oxygen species (ROS) detection: The intracellular ROS production was detected by using 2,7-dichlorofluorescein diacetate (DCFH-DA). Firstly, 4T1 cells in the logarithmic growth phase were seeded into confocal dishes at a density of 1×10^5 cells per dish and cultured overnight in a humidified incubator at 37°C with 5% CO_2 to allow adherence. Subsequently, the cells were treated with 10 μg/mL TFN-Me NPs, Nap-TFN-Me NPs and TPETPA-TFN NPs, respectively. After 8 h of incubation, the cells were washed three times with PBS, followed by incubation with 1 μM DCFH-DA for 20 min. The cells in the light group were irradiated with a white light laser (50 mW/cm^2) for 10 min, while the dark control group was maintained under light proof conditions. Finally, intracellular fluorescence intensity was observed by confocal laser scanning microscopy (CLSM) to evaluate the ROS generation levels in the cells.

Live-dead cell staining: 4T1 cells in the logarithmic growth phase were seeded into confocal dishes at a density of 1×10^5 cells per dish and cultured overnight in a humidified incubator at 37°C with 5% CO_2 to allow adherence. The cells were then treated with 10 μg/mL TFN-Me NPs, Nap-TFN-Me NPs and TPETPA-TFN NPs, respectively. After 8 hours of co-culture, cells in the light group were irradiated with white light laser (50 mW/cm^2) for 10 min, while the dark control group was maintained under light proof conditions. Thereafter, the cells were washed three times with PBS, followed by staining with a mixture of 1 μM Calcein-AM and 1 μM PI in assay buffer for 20 min at 37°C. After incubation, residual dyes were removed by three addition PBS washes.

Cell viability: Cell viability was determined by MTT colorimetric assay, which is based on the reduction of 3-(4,5-dimethylthiazol-2-yl)-2,5-diphenyltetrazolium bromide (MTT) to formazan by succinate dehydrogenase in the mitochondria of viable cells. Firstly, 100 μL of cell suspension (5000 cells/well) was added to the 96 well plate and cultured overnight in a humidified incubator containing 5% CO_2 at 37°C for adherence. Then DMEM medium (100 μL) containing different concentrations of nanoparticles (0-50 μg/mL) was added to 96 well plates for 24 hours of co-culture. Then, 20 μL of MTT (5 mg/mL) was added to each well containing the above-mentioned cells, continue

culturing for 4 hours at 37 °C under a 5% CO_2 atmosphere in the dark. Subsequently, aspirated and discarded the liquid, 150 μL of DMSO was added to each well, and measured the absorbance (OD) of each well using a microplate reader. The average value of duplicate wells was taken as the OD value for each sample concentration. The calculation formula for cell viability is as follows:

Cell viability (%) = OD value of sample group / OD value of blank group × 100%

Hemolysis experiments: Blood was taken from the mouse canthus, first centrifuged to remove the supernatant, and then PBS was added for washing to obtain pure RBCs. Thereafter, mix 100 μL of red blood cell suspension with 2% Triton-100, normal saline and TFN-Me NPs or TPETPA-TFN NPs (6.25, 12.5, 25, 50 μg/mL) containing different concentrations of 900 μL. Incubate at 37°C for 3 hours and centrifuge all samples at 5,000 rpm for 10 min. Finally, the sample was photographed, and the absorbance of the supernatant at 540 nm was detected by a microplate reader to calculate the hemolysis rate.

Serum biochemistry assay: To evaluate heart, liver and kidney function, fresh whole blood was collected from healthy female BALB/c mice in 1.5 mL centrifuge tube on ten days post-injection. After standing at room temperature for 2 h, the whole blood was centrifuging at 3000 rpm for 40 min to obtain serum for subsequent serum biochemical index detection. Serum biochemical index: alanine aminotransferase (ALT), aspartate aminotransferase (AST), blood urea nitrogen (BUN), uric acid (UA), creatine kinase (CK), lactate dehydrogenase (LDH).

Subcutaneous tumor bearing mouse model: All the animal studies according to the Guide for Care and Use of Laboratory Animals, approved by the Animal Experimentation Ethics Committee of Southern Medical University (00323972). The 4T1 subcutaneous tumor model was established by subcutaneous (s.c.) injection of 100 μL of 4T1 cell suspension (approximately 5×10^6 cells) into the flank regions of female BALB/c mice (n = 3).

In vivo fluorescence imaging of subcutaneous tumors: Mice with subcutaneous tumor volume of about 200 mm^3 were intravenously injected with TFN-Me NPs and

TPETPA-TFN NPs, and intravital fluorescence imaging was performed using a mouse intravital imaging system at 1h, 4h, 8h, 12h, 24h, 36h, and 48h after injection.

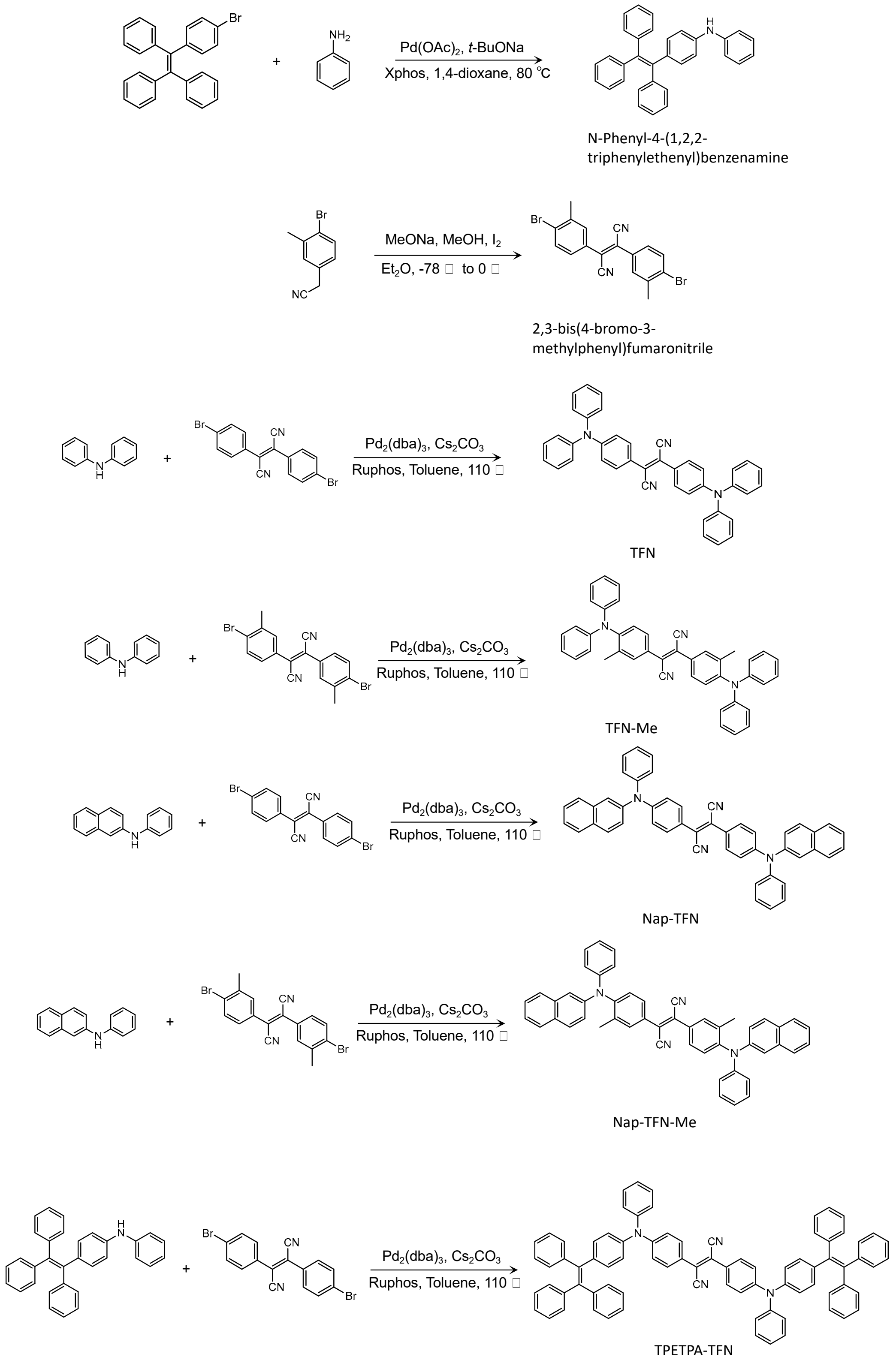

Scheme S1. The synthetic routes to TFN, TFN-Me, Nap-TFN, Nap-TFN-Me and TPETPA-TFN.

TFN, Nap-TFN, and TPETPA-TFN were synthesized according to reported literature [1,2].

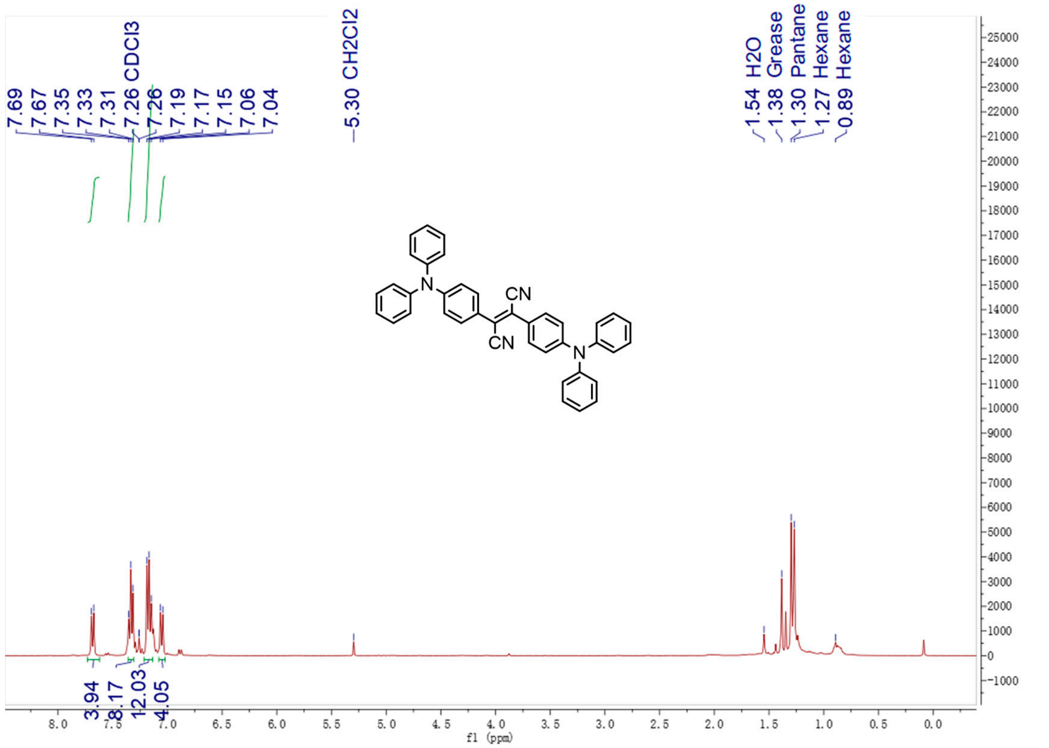

Figure S1. ^{1}H NMR spectrum of TFN in $CDCl_3$.

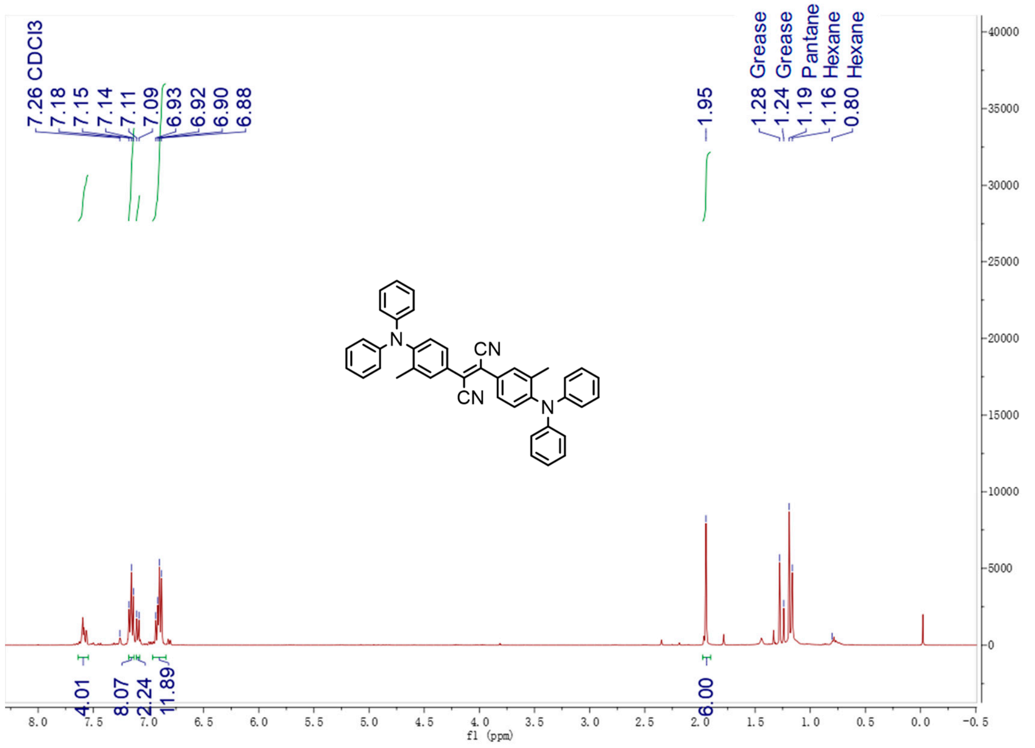

Figure S2. ^{1}H NMR spectrum of TFN-Me in $CDCl_3$.

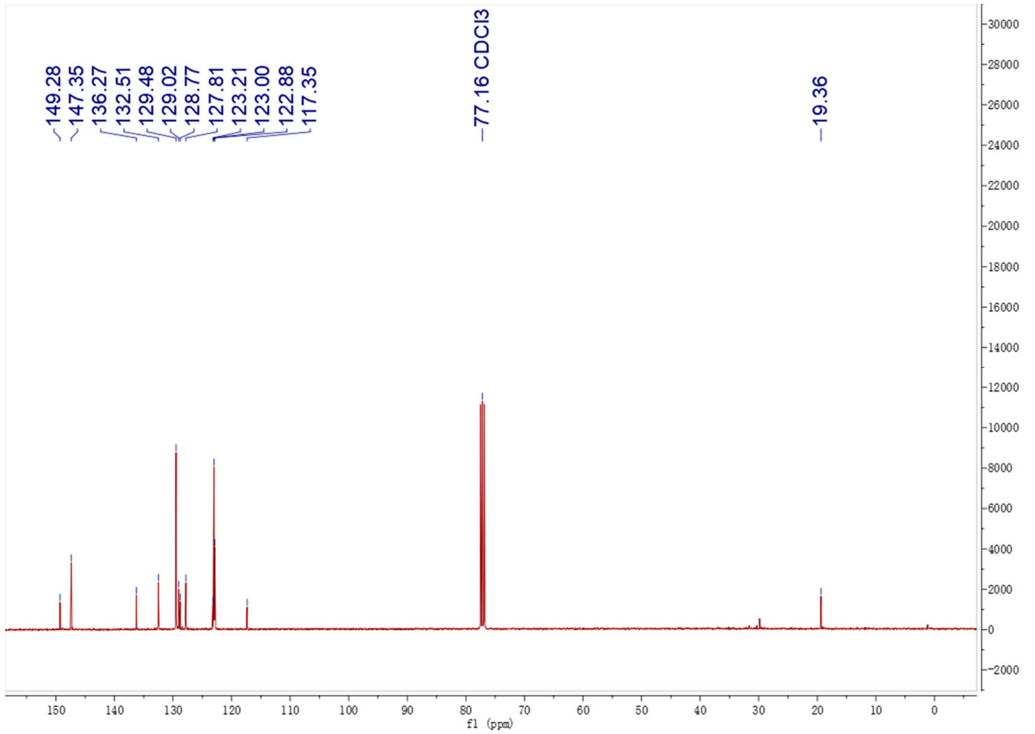

Figure S3. ^{13}C NMR spectrum of TFN-Me in $CDCl_3$.

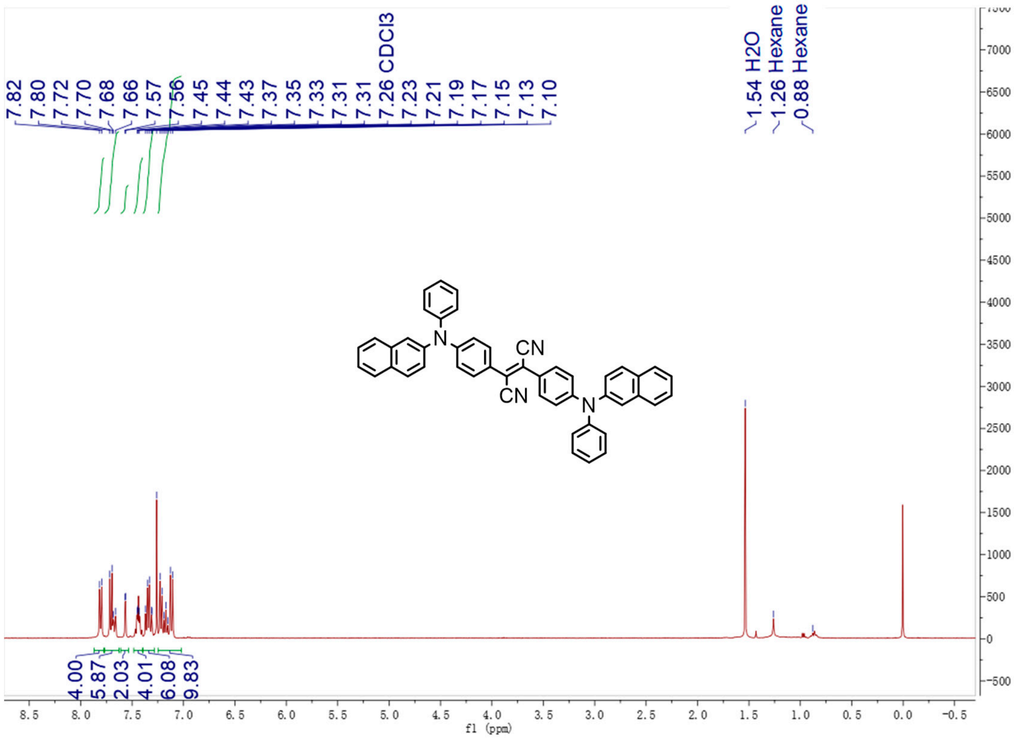

Figure S4. ^{1}H NMR spectrum of Nap-TFN in $CDCl_3$.

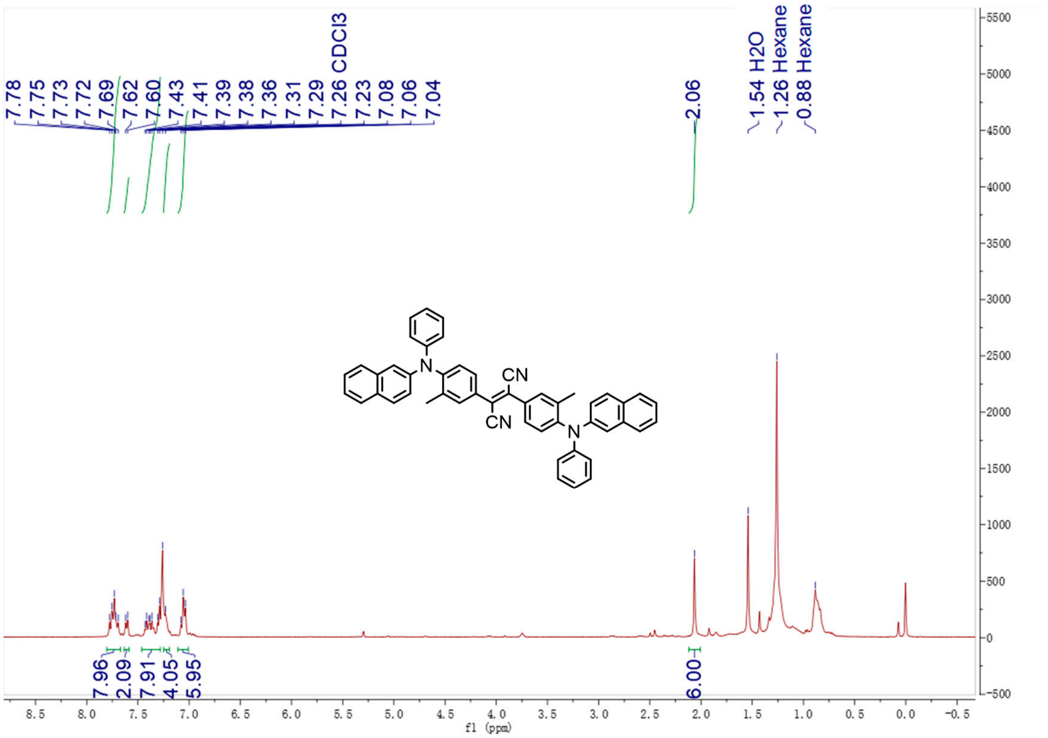

Figure S5. ^{1}H NMR spectrum of Nap-TFN-Me in $CDCl_3$.

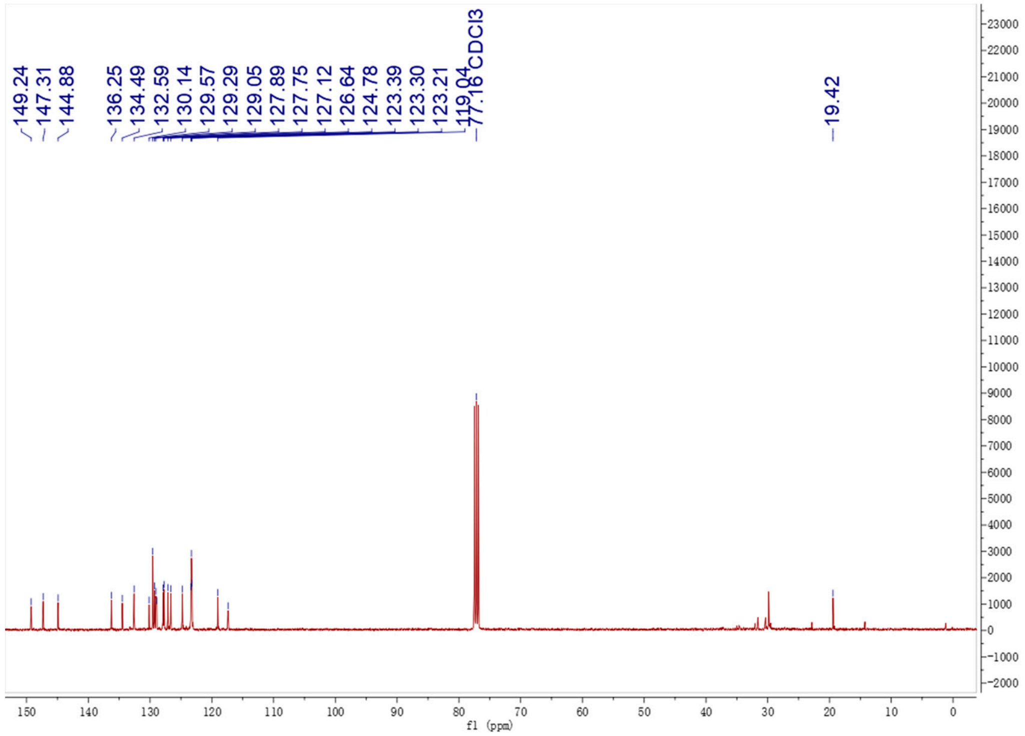

Figure S6. ^{13}C NMR spectrum of Nap-TFN-Me in $CDCl_3$.

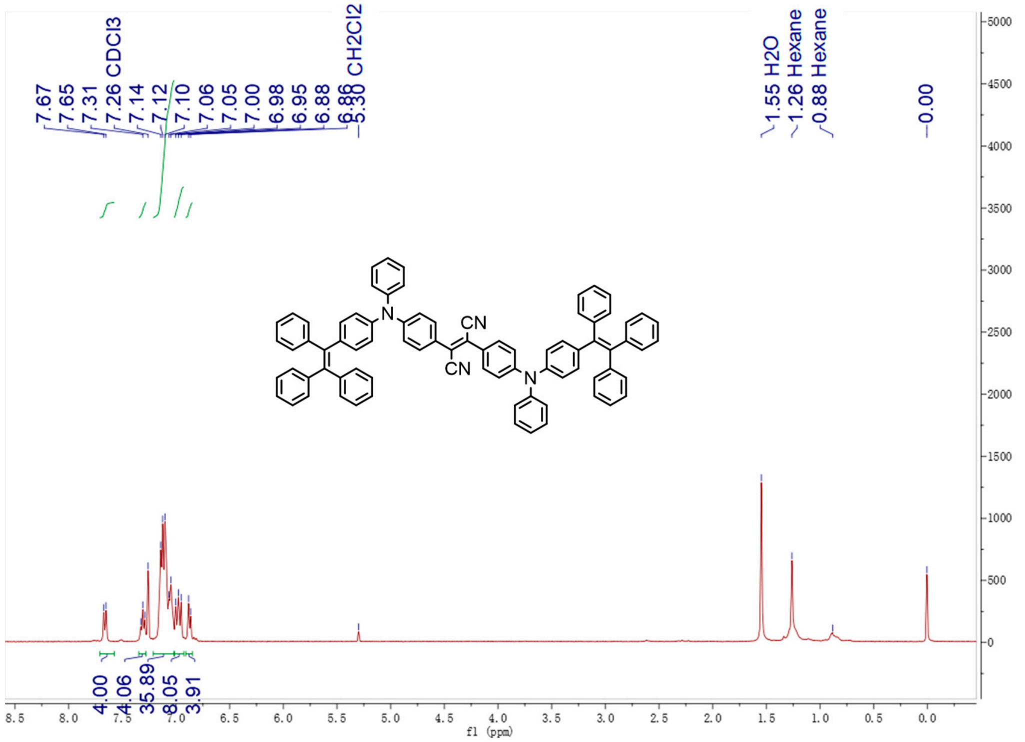

Figure S7. ^{1}H NMR spectrum of TPETPA-TFN in $CDCl_3$.

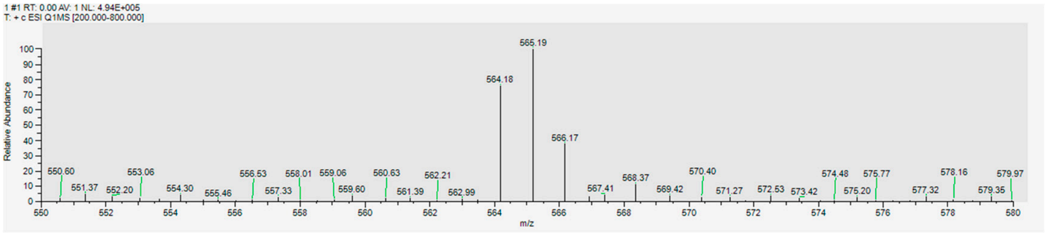

Figure S8. Mass spectrum of TFN.

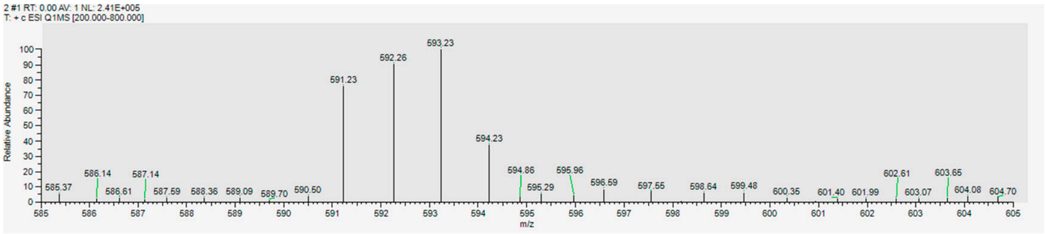

Figure S9. Mass spectrum of TFN-Me.

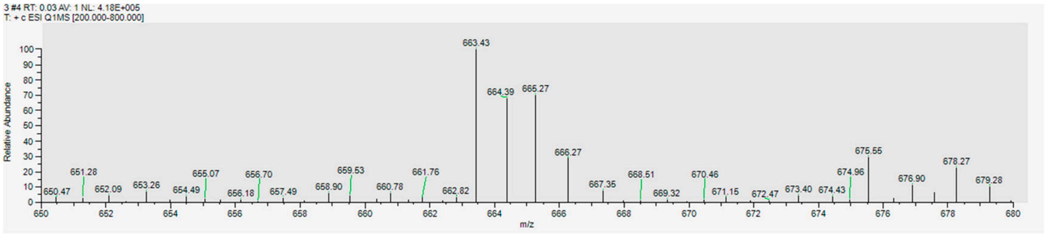

Figure S10. Mass spectrum of Nap-TFN.

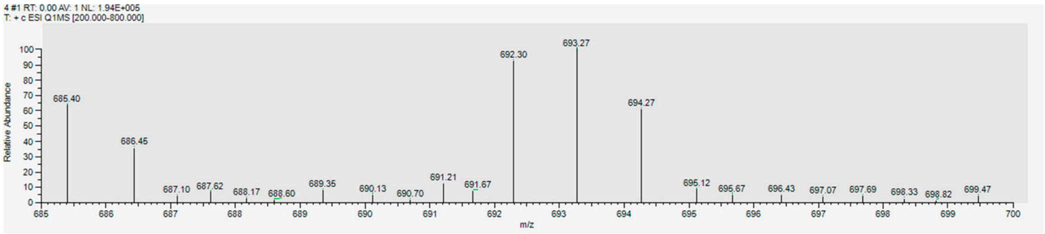

Figure S11. Mass spectrum of Nap-TFN-Me.

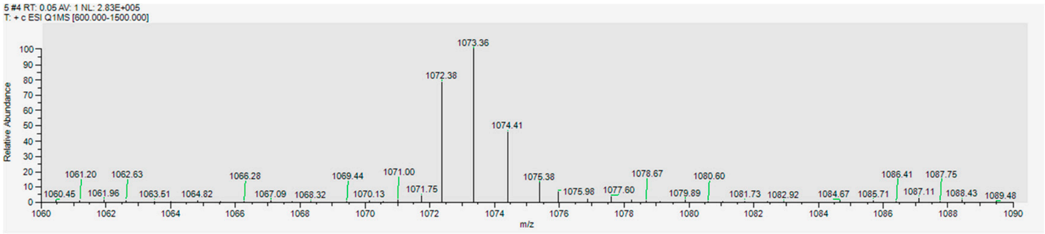

Figure S12. Mass spectrum of TPETPA-TFN.

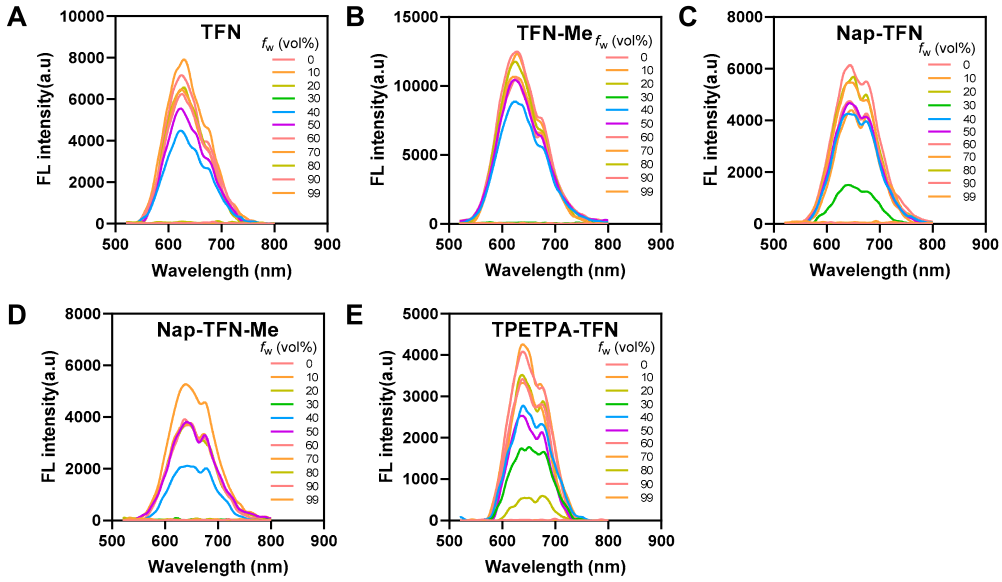

Figure S13. Fluorescence spectra of TFN (A), TFN-Me (B), Nap-TFN (C), Nap-TFN-Me (D) and TPETPA-TFN (E) (10 μM) in different DMSO/water mixtures.

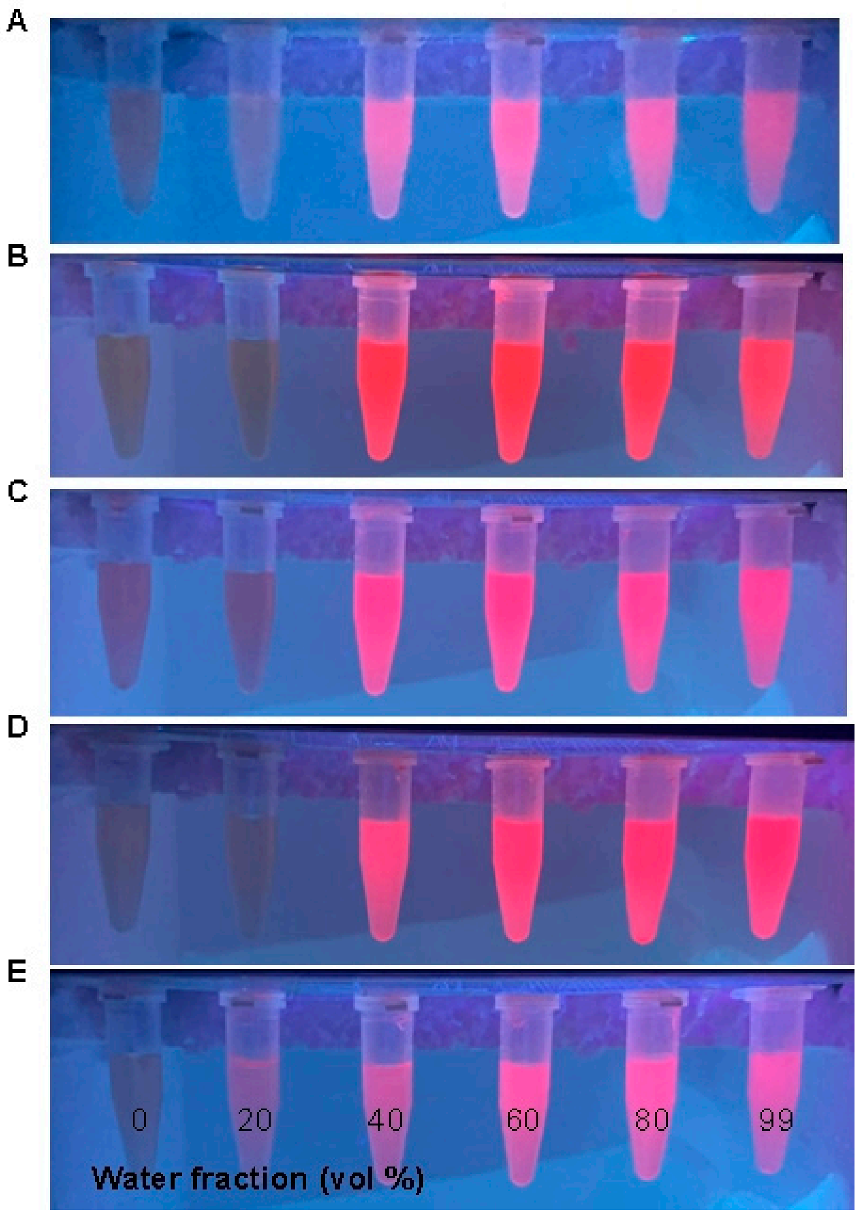

Figure S14. The fluorescence photographs of TFN (A), TFN-Me (B), Nap-TFN (C), Nap-TFN-Me (D) and TPETPA-TFN (E) in DMSO/water mixtures with different water contents under UV light irradiation. λ_{ex} = 365 nm.

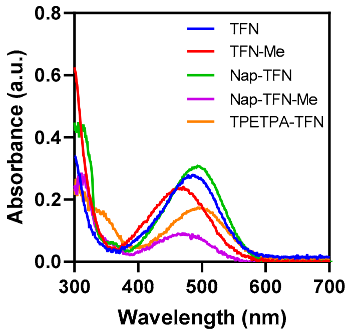

Figure S15. Absorption spectra of TFN, TFN-Me, Nap-TFN, Nap-TFN-Me and TPETPA-TFN (10 μM) in DMSO solution.

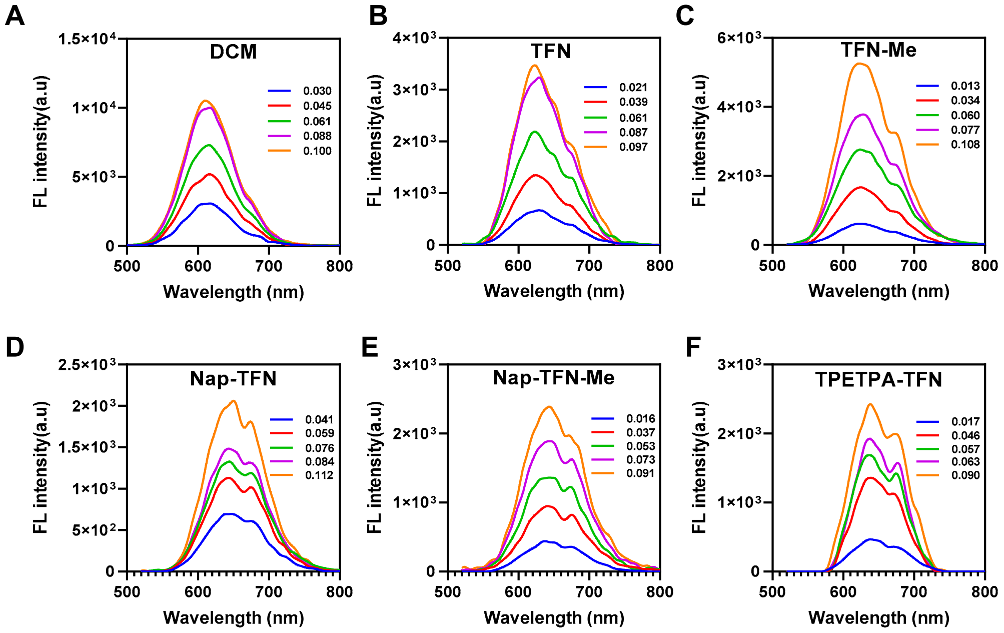

Figure S16. Fluorescence spectra of DCM (A) in methanol, TFN (B), TFN-Me (C), Nap-TFN (D), Nap-TFN-Me (E) and TPETPA-TFN (F) in the DMSO/water (1/99, *v*/*v*) solution with different absorbance values.

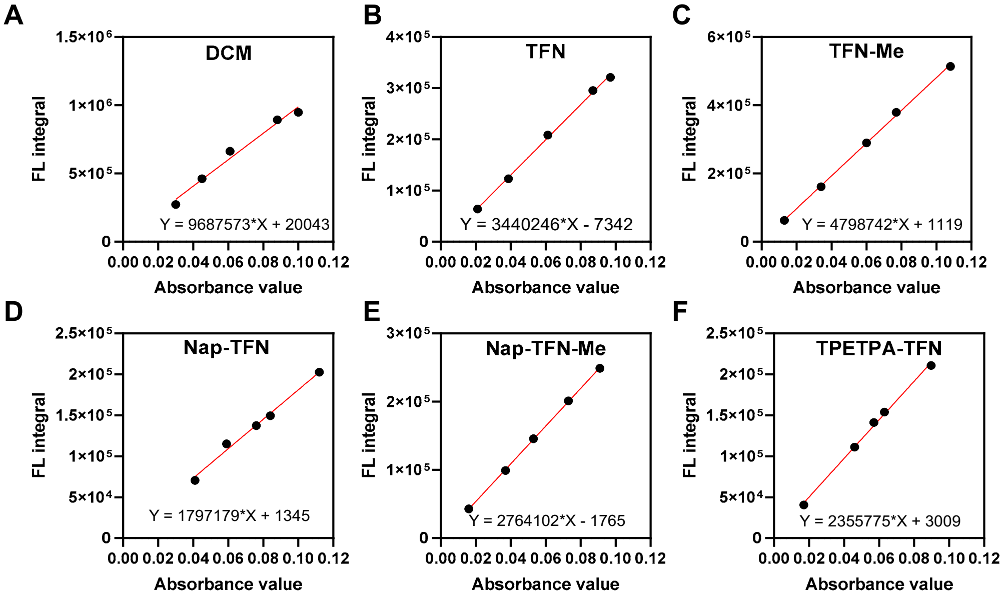

Figure S17. The relationship between the absorbance value and the peak area of fluorescence spectrum measured by DCM (A), TFN (B), TFN-Me (C), Nap-TFN (D), Nap-TFN-Me (E) and TPETPA-TFN (F), and their linear regression fitting curve, respectively.

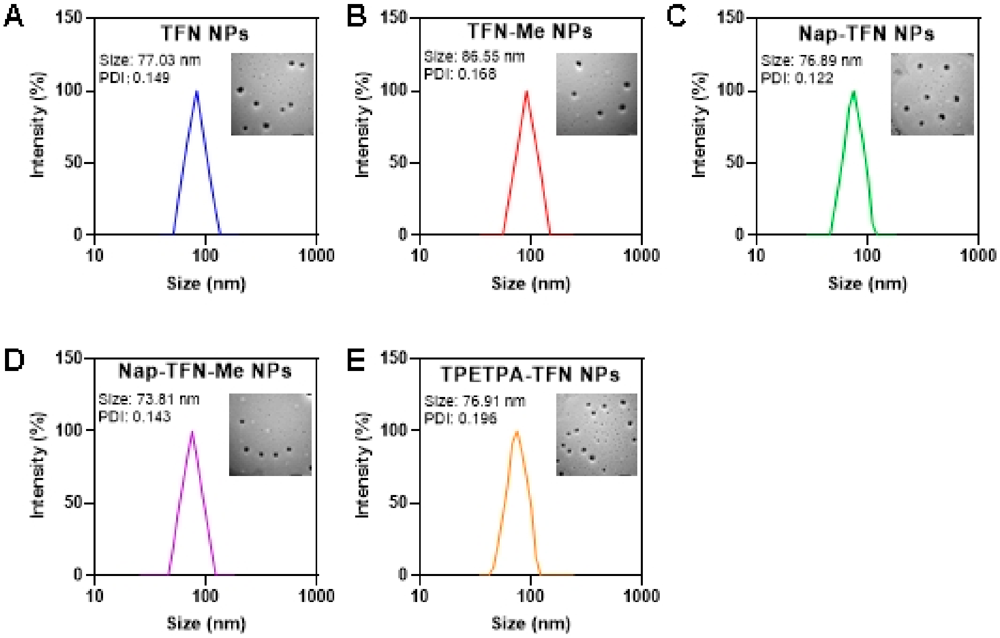

Figure S18. Individual hydrodynamic size distribution and the TEM images (inset photos) of TFN NPs (A), TFN-Me NPs (B), Nap-TFN NPs (C), Nap-TFN-Me NPs (D) and TPETPA-TFN NPs (E). Scale bars = 200 nm.

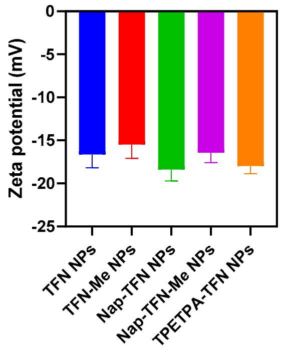

Figure S19. The zeta potential of TFN NPs, TFN-Me NPs, Nap-TFN NPs, Nap-TFN-Me NPs and TPETPA-TFN NPs in water. Error bars: mean ± SD (n = 3).

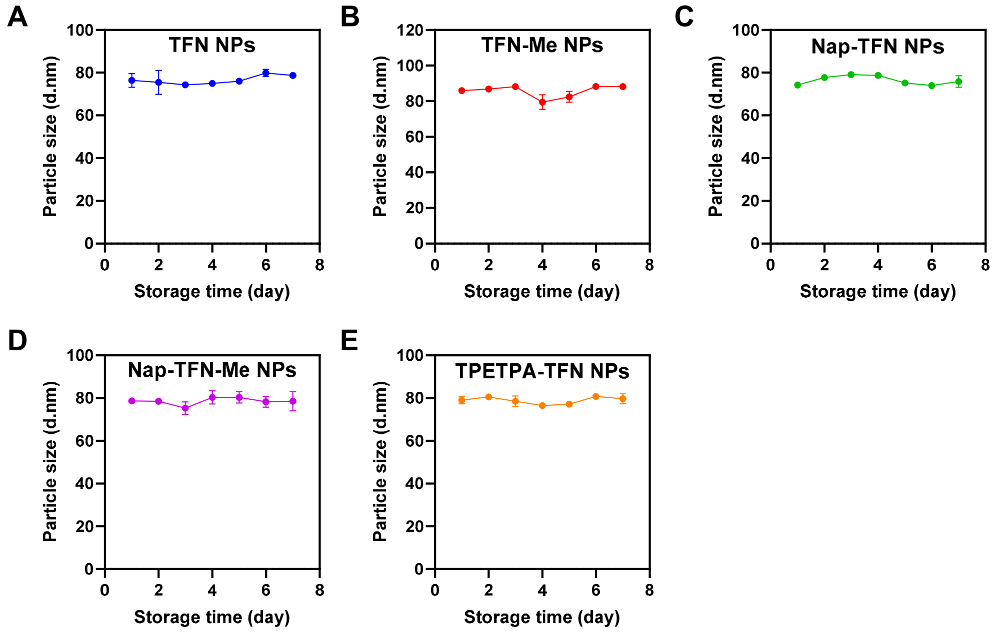

Figure S20. The size changes of TFN NPs (A), TFN-Me NPs (B), Nap-TFN NPs (C), Nap-TFN-Me NPs (D) and TPETPA-TFN NPs (E) within seven storage days, measured by DLS. Error bars: mean ± SD (n = 3).

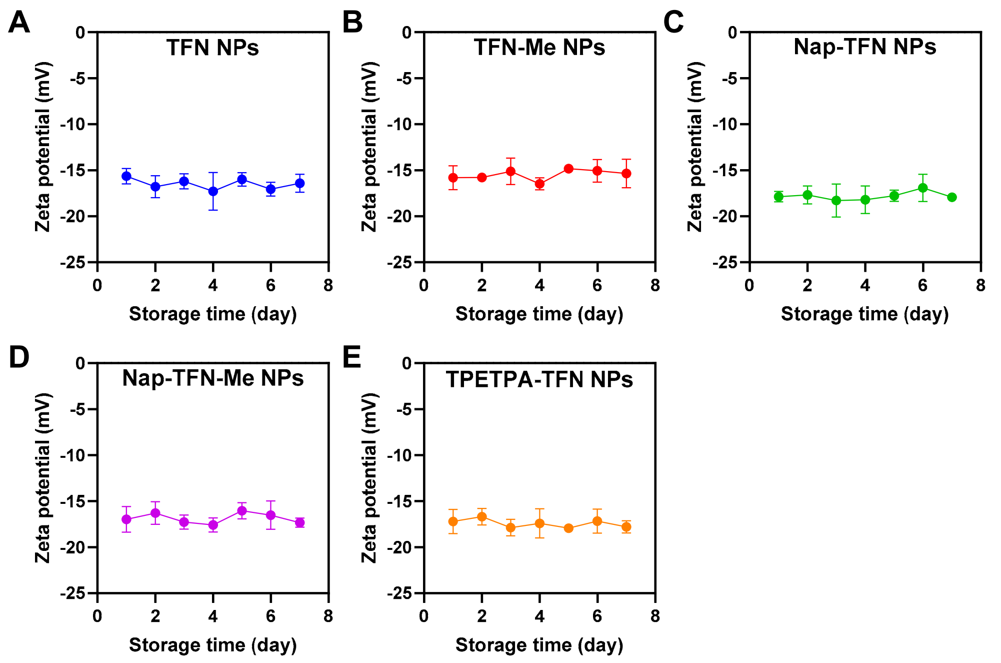

Figure S21. The zeta potential changes of TFN NPs (A), TFN-Me NPs (B), Nap-TFN NPs (C), Nap-TFN-Me NPs (D) and TPETPA-TFN NPs (E) within seven storage days. Error bars: mean ± SD (n = 3).

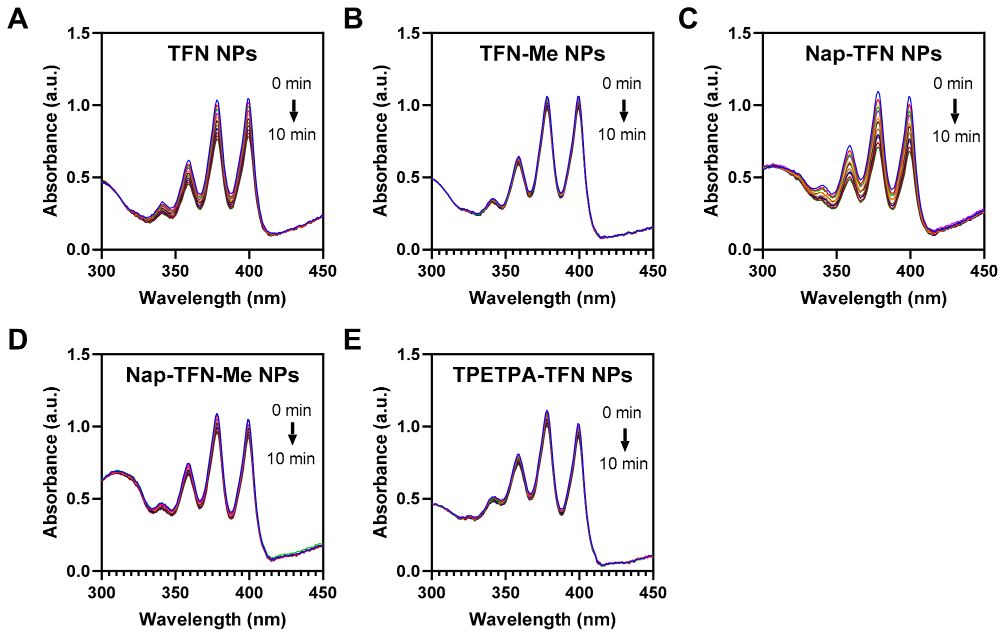

Figure S22. Absorption spectra of mixed solutions of ABDA (100 μM) with TFN NPs (A), TFN-Me NPs (B), Nap-TFN NPs (C), Nap-TFN-Me NPs (D) and TPETPA-TFN NPs (E) in water under 50mW/cm^2 white light irradiation at different times.

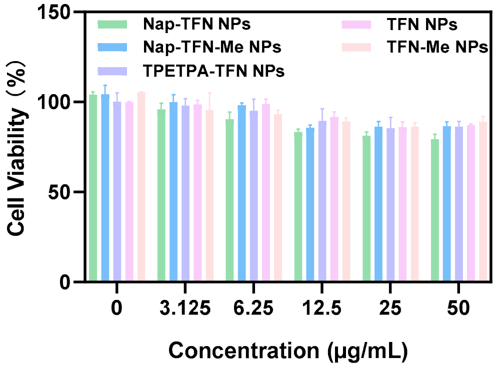

Figure S23. 4T1 cell viabilities after treating with different concentrations of TFN NPs, TFN-Me NPs, Nap-TFN NPs, Nap-TFN-Me NPs and TPETPA-TFN NPs upon white light irradiation (100 mW/cm^2) for 5 min. Error bars: mean ± SD (n = 5).

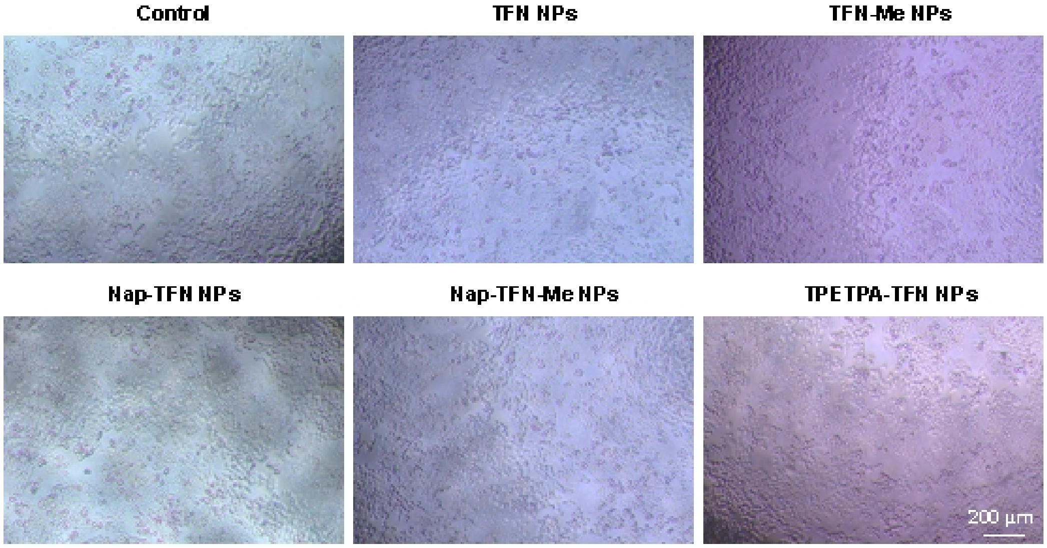

Figure S24. The 4T1 cell images observed by microscope upon treatment with TFN NPs, TFN-Me NPs, Nap-TFN NPs, Nap-TFN-Me NPs and TPETPA-TFN NPs (100 μg/mL). Scale bars = 200 μm.

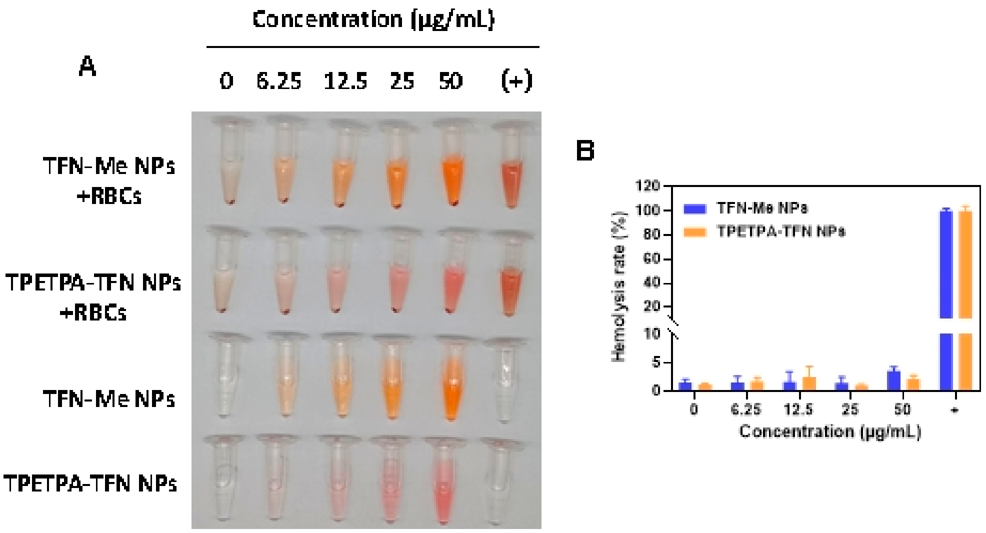

Figure S25. (A) Hemolysis rate of red blood cells after being treated with TFN-Me NPs and TPETPA-TFN NPs at a different concentration from 0 to 50 μg/mL for 3 h at 37 °C, using Triton X-100 as a positive control and nanoparticles in PBS without RBCs as a negative control. Error bars: mean ± SD (n = 4). (B) The quantified analysis.

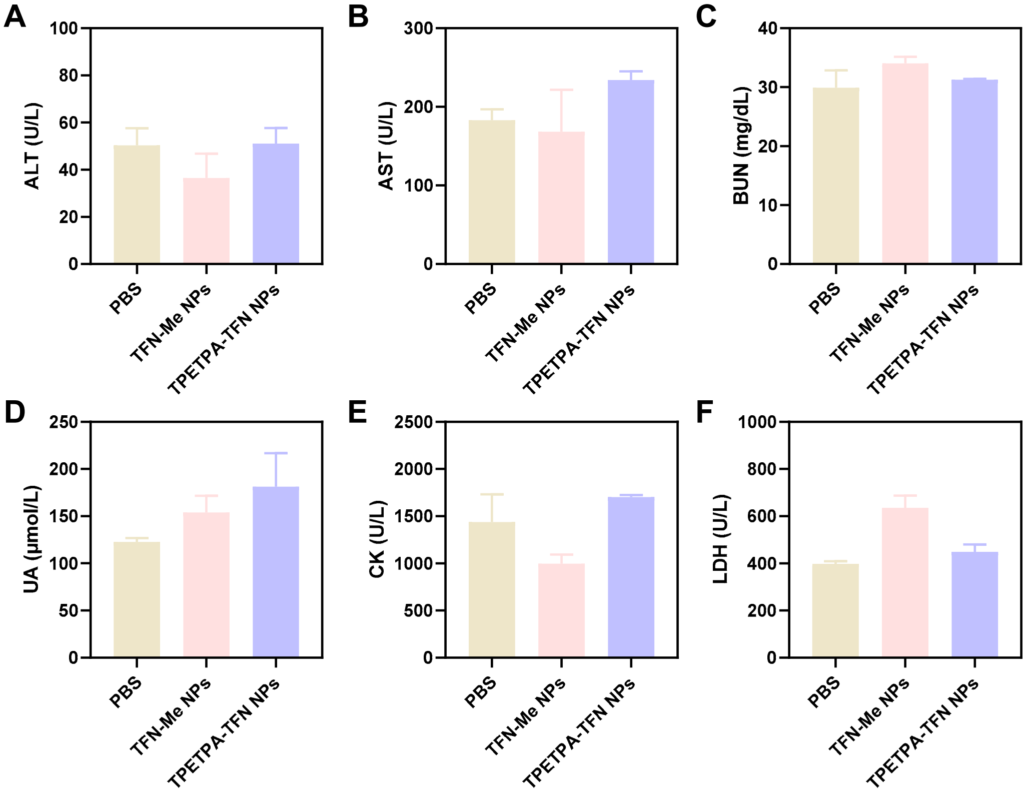

Figure S26. Blood biochemistry of mice on ten days post-injection of PBS, TFN-Me NPs and TPETPA-TFN NPs: alanine aminotransferase (ALT) (A), aspartate aminotransferase (AST) (B), blood urea nitrogen (BUN) (C), uric acid (UA) (D), creatine kinase (CK) (E), lactate dehydrogenase (LDH) (F). Error bars: mean ± SD (n = 3).

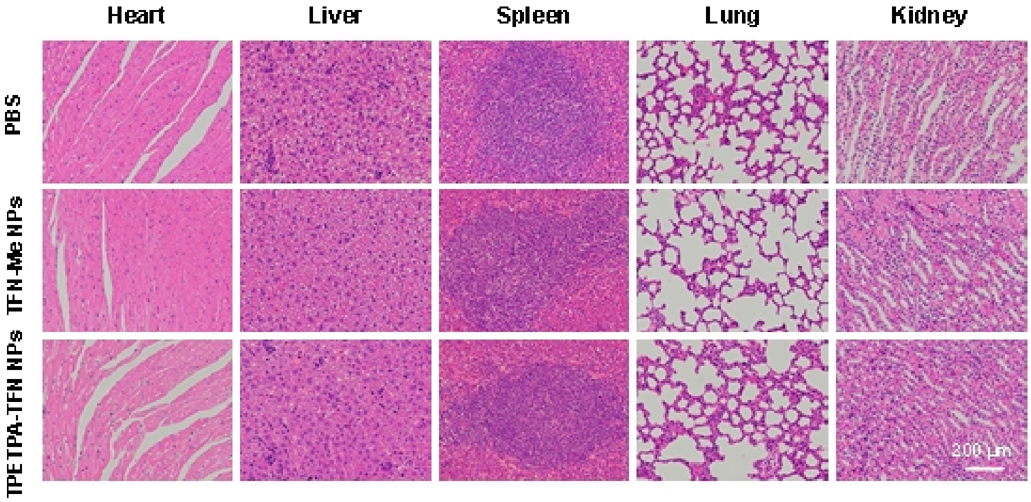

Figure S27. H&E staining of normal organ tissue sections of different groups of mice after treatment. Scale bars = 200 μm.

Reference:

1. Wang, Q.; Li, C.B.; Song, Y.C.; Shi, Q.K.; Li, H.; Zhong, H.; Wang, J.G.; Hu, F. Acene enlargement for absorption red-shifting and photosensitization enhancement of photosensitizers with aggregation-induced emission. *Chem. Sci*. **2023**, *14*, 684-690.

2. Geng, J.L.; Li, K.; Qin, W.; Ma, L.; Gahik, G.G.; Tang, B.Z.; Liu, B. Eccentric Loading of Fluorogen with Aggregation-Induced Emission in PLGA Matrix Increases Nanoparticle Fluorescence Quantum Yield for Targeted Cellular Imaging. *Small*. **2013**, *9*, 2012-2019.
